# Valrubicin-loaded immunoliposomes for specific vesicle-mediated cell death in the treatment of hematological cancers

**DOI:** 10.1038/s41419-024-06715-5

**Published:** 2024-05-11

**Authors:** Aleksandra Georgievski, Pierre-Simon Bellaye, Benjamin Tournier, Hélène Choubley, Jean-Paul Pais de Barros, Michaële Herbst, Arnaud Béduneau, Patrick Callier, Bertrand Collin, Frédérique Végran, Paola Ballerini, Carmen Garrido, Ronan Quéré

**Affiliations:** 1 Center for Translational and Molecular Medicine, UMR1231 Inserm/Université de Bourgogne, Dijon, France; 2 LipSTIC Labex, Dijon, France; 3 Plateforme d’imagerie et de radiothérapie précliniques, Centre Georges François Leclerc-Unicancer, Dijon, France; 4 Service de Pathologie, Plateforme de génétique somatique des cancers de Bourgogne, CHU Dijon-Bourgogne, Dijon, France; 5 Plateforme DiviOmics, UMS58 Inserm BioSanD, Université de Bourgogne, Dijon, France; 6 Laboratoire Interdisciplinaire Carnot de Bourgogne, UMR6303 CNRS/Université de Bourgogne, Dijon, France; 7 Université de Franche-Comté, EFS, Inserm, UMR1098 RIGHT, Besançon, France; 8 Laboratoire de Génétique Chromosomique et Moléculaire, CHU Dijon-Bourgogne, Dijon, France; 9 Centre Georges François Leclerc-Unicancer, Dijon, France; 10 Laboratoire d’Hématologie, Assistance Publique-Hôpitaux de Paris, Hôpital Armand Trousseau, Paris, France; 11 Label of excellence from la Ligue Nationale contre le Cancer, Paris, France

**Keywords:** Leukaemia, Lymphoma, Drug development, Preclinical research

## Abstract

We created valrubicin-loaded immunoliposomes (Val-ILs) using the antitumor prodrug valrubicin, a hydrophobic analog of daunorubicin. Being lipophilic, valrubicin readily incorporated Val-lLs that were loaded with specific antibodies. Val-ILs injected intravenously rapidly reached the bone marrow and spleen, indicating their potential to effectively target cancer cells in these areas. Following the transplantation of human pediatric B-cell acute lymphoblastic leukemia (B-ALL), T-cell acute lymphoblastic leukemia (T-ALL), or acute myeloid leukemia (AML) in immunodeficient NSG mice, we generated patient-derived xenograft (PDX) models, which were treated with Val-ILs loaded with antibodies to target CD19, CD7 or CD33. Only a small amount of valrubicin incorporated into Val-ILs was needed to induce leukemia cell death in vivo, suggesting that this approach could be used to efficiently treat acute leukemia cells. We also demonstrated that Val-ILs could reduce the risk of contamination of CD34^+^ hematopoietic stem cells by acute leukemia cells during autologous peripheral blood stem cell transplantation, which is a significant advantage for clinical applications. Using EL4 lymphoma cells on immunocompetent C57BL/6 mice, we also highlighted the potential of Val-ILs to target immunosuppressive cell populations in the spleen, which could be valuable in impairing cancer cell expansion, particularly in lymphoma cases. The most efficient Val-ILs were found to be those loaded with CD11b or CD223 antibodies, which, respectively, target the myeloid-derived suppressor cells (MDSC) or the lymphocyte-activation gene 3 (LAG-3 or CD223) on T4 lymphocytes. This study provides a promising preclinical demonstration of the effectiveness and ease of preparation of Val-ILs as a novel nanoparticle technology. In the context of hematological cancers, Val-ILs have the potential to be used as a precise and effective therapy based on targeted vesicle-mediated cell death.

## Introduction

Advancements in the treatments available for acute lymphoblastic leukemia (ALL), acute myeloid leukemia (AML), and lymphoma have led to increased complete remission rates, with 5-year survival rates exceeding 80% for ALL, 50% for AML and 70% for lymphoma [1–10]. Still, there remains a need to develop new treatment strategies aimed at reducing the intensity of therapies and enhancing patient prognosis following a relapse. Unilamellar vesicles (UVs) are vesicular structures characterized by a lipid bilayer, and their structure enables them to transport a wide range of hydrophilic and hydrophobic compounds. In particular, liposomes have been extensively employed as pharmaceutical carriers for various drugs, especially in cancer treatment [11–17]. Since their discovery over 50 years ago [18], liposomes have evolved into a promising tool in the fields of medicine, biology, and chemistry. This is a result of their biocompatibility and their capacity to encapsulate and deliver a diverse array of drugs for therapeutic purposes [11–17].

Drug-loaded liposomes can be directed to tumors using passive targeting, a method that has been employed in the treatment of hematological cancers. For example, “passive liposomes” used to treat hematological malignancies include liposomal daunorubicin, which has been developed for the treatment of relapsed and refractory AML [19–21]. In addition, vincristine sulfate liposome injection has been developed to enhance the pharmacokinetics and pharmacodynamics of vincristine [22]. This formulation has been utilized in clinical settings to treat patients with relapsed and refractory lymphoma [23], as well as young patients with refractory solid tumors or leukemia [22, 24]. These advancements in liposomal drug delivery have shown promise in improving the treatment outcomes for patients with various hematological malignancies.

“Active liposomes” constitute another approach involving the attachment of specific ligands to the surface of liposomes to bind to particular antigens on target cells [11–17]. To actively target specific cells, a variety of ligands, including antibodies, proteins, carbohydrates, and even aptamers, have been attached to the surface of liposomes [11–17]. Within the realm of immunotherapy, immunoliposomes (ILs) have emerged as a novel strategy that has been extensively investigated in preclinical solid cancer models, showing promising results [11–17]. Further experiments with mouse models have demonstrated that ILs encapsulating the anthracycline doxorubicin can target cancer cells and enhance the immunotherapeutic effect by reshaping the immunosuppressive tumor microenvironment [25–28]. In the field of hematology, therapeutic ILs targeting CD20 [29, 30] and CD19 [31–34] have been developed to treat B-cell lymphoma both in vitro and in vivo using xenograft models. ILs-αCD19, containing imatinib, have been shown to efficiently kill Philadelphia chromosome-positive ALL cells in vitro [35]. The delivery of PEGylated liposomal doxorubicin using bispecific antibodies has shown improved efficacy in models of high-risk childhood leukemia [36]. Additionally, antibody fragment-decorated liposomal conjugates have successfully targeted Philadelphia-like ALL [37]. Nanoparticle-mediated targeting of the fusion gene *RUNX1::ETO* has also been reported in AML [38]. These are all exciting developments in the application of liposomal drug delivery for treating various hematological malignancies.

The majority of established ILs have relied on encapsulating hydrophilic drugs within their internal aqueous compartments [29–35, 39, 40]. However, in this particular study, our objective was to create ILs loaded with valrubicin, a hydrophobic analog of doxorubicin. Valrubicin is recognized for its use in intravesical chemotherapy for carcinoma in situ of the bladder [41–45]. Given its lipophilic nature, valrubicin was found to integrate effectively into the liposomes. We thus investigated the efficacy of these liposomes with valrubicin incorporated into the lipids, which we called Val-ILs, using in vitro and in vivo leukemia and lymphoma models. Our primary goal was to explore this novel nanoparticle technology designed to induce specific vesicle-mediated cell death for the treatment of hematological cancers. This approach offers a promising avenue for the targeted treatment of these malignancies.

## Results

### Development of ILs incorporated with valrubicin

**Fig. 1:**
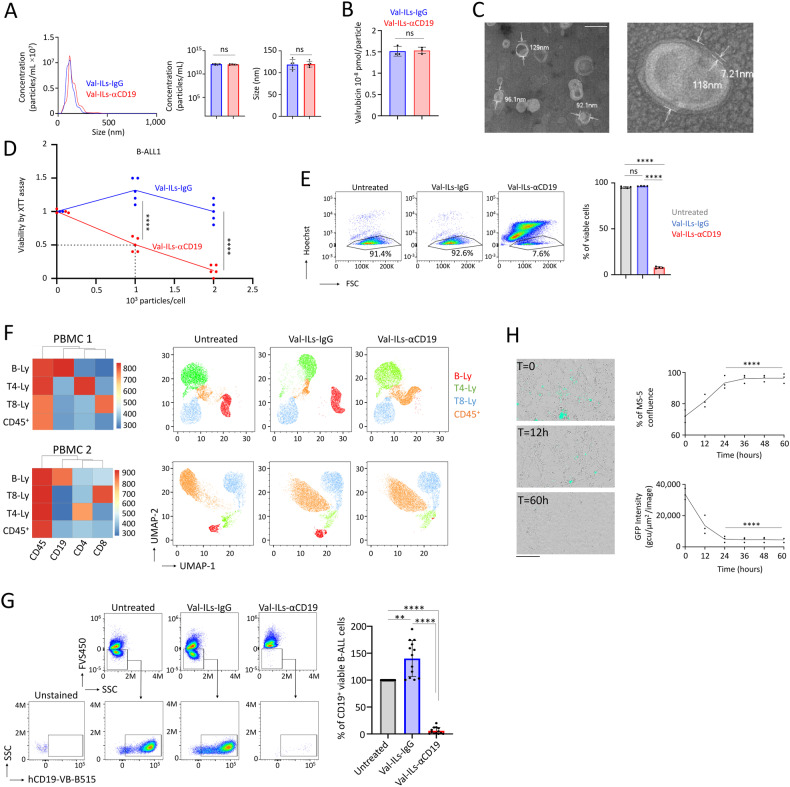
Val-ILs-αCD19 efficiently induces the death of human CD19^+^ cells. **A** Quantification and size of Val-ILs measured by NTA, *n* = 5 biological replicates. **B** HPLC–MS/MS for the quantification of Valrubicin on Val-ILs-IgG and Val-ILs-αCD19, *n* = 3 biological replicates. **C** Representative pictures of Val-ILs-αCD19 observed by TEM, magnification ×94K, white scale represents 200 nm. **D** Viability assessed on CD19-expressing BALL-1 cells by XTT assay, following a single treatment, on day 0 with 1000 or 2000 particles/cell. The IC50 measured at 1000 particles/cell. XTT assay performed after 72 h of treatment, normalized to untreated, *n* = 5 biological replicates. **E** Viability assessed by flow cytometry on BALL-1 cells, treated with Val-ILs-IgG or Val-ILs-αCD19 (2000 particles/cell). Flow cytometry was performed after 72 h of treatment. Val-ILs-αCD19 efficiently affected the viability of BALL-1 cells, but this was not the case for Val-ILs-IgG. Examples of cytometry dataset and statistics, *n* = 4 biological replicates. **F** Flow cytometry shows how Val-ILs-αCD19 eliminates the population of B-lymphocytes expressing CD19 among human peripheral blood mononuclear cells (PBMC) after 48 h of in vitro exposure with 2000 particles/cells. Flow cytometry UMAP data frames are shown for two PBMC samples. B-lymphocytes (B-Ly), T4-lymphocytes (T4-Ly), T8-lymphocytes (T8-Ly), and the remaining hematopoietic cells (CD45^+^). **G** Val-ILs-αCD19 eliminates CD19^+^ B-ALL cells isolated from human BM at diagnosis after 48 h of ex vivo exposure with 2000 particles/cell. Flow cytometry dataset of one representative primary B-ALL sample. Statistics show that Val-ILs-αCD19 efficiently eliminates >90% of the CD19^+^ B-ALL cells, while Val-ILs-IgG had no effect. The percentage of viable cells is normalized to untreated controls, *n* = 13 B-ALL patients. **H** Val-ILs-αCD19 affects B-ALL cells but not the stromal mesenchymal supportive MS-5 cells. Pictures at different times show the disappearance of GFP^+^ cells following treatment with 2000 particles/B-ALL cells. Black scale bars represent 200 µm. The percentage of MS-5 cells’ confluence over time indicates that Val-ILs-αCD19 did not have an impact on the growth of the supportive MS-5 cells. GFP intensity was measured over time, showing that Val-ILs-αCD19 affected the viability of B-ALL cells within 24 h. Data are shown as means ± SD, *n* = 3 biological replicates. In this figure, data are shown as means ± SD, **A**, **B**, **D** *P* value measured by two-tailed unpaired Student’s *t*-test; ****P < 0.0001; ns, non-significant. **E**, **G**, **H** *P* value measured by one-way ANOVA with Tukey’s multiple comparison test; **P < 0.01; ****P < 0.0001; ns, non-significant.

In our formulation, we used a mixture of l-α-phosphatidylcholine, cholesterol, DSPE-PEG-square (2000), and DSPE-PEG-NHS (5000), which were prepared in chloroform with specific molar ratios of 100:37:1.5:0.2. Valrubicin reconstituted in dimethyl sulfoxide (DMSO) was added at 1 mM. The liposomes were then created using a thin film hydration method in PBS 1×, followed by sonication. The CD19 antigen is a transmembrane protein specific to B-cells and often utilized as a target for B-cell leukemia and lymphoma [46–48]. Once the unilamellar vesicles (UVs) were stabilized, we incorporated an anti-CD19 antibody (Val-ILs-αCD19) or the IgG isotype control (Val-ILs-IgG) just before the purification process via dialysis. Nanoparticle tracking analysis (NTA) found that Val-ILs, with an average diameter of 115 nm, correspond more to large UVs (ranging from 100 nm to 1 µm), and their concentration ranged from 0.8 × 10^12^ to 1.5 × 10^12^ particles per milliliter (Fig. 1A). Through Western blot analysis, we determined that approximately 50% of the antibodies were bound to the Val-ILs, and dialysis successfully removed more than 95% of the unbound antibodies (Fig. S1A). Additionally, the presence of the anti-CD19 antibody or IgG isotype control was confirmed through flow cytometry analysis (Fig. S1B). High-performance liquid chromatography coupled with mass spectrometry (HPLC-MS/MS) was used to quantify the valrubicin content in both Val-ILs-αCD19 and Val-ILs-IgG, revealing that each contained 1.52 × 10^−8^ pmol of valrubicin per particle (Fig. 1B). The observation that approximately 58% of the valrubicin used in the preparation of Val-ILs was successfully incorporated is a significant finding. Additionally, the successful removal of all non-encapsulated valrubicin through dialysis confirms that the drug was completely integrated into the Val-ILs, and there was no contamination from unincorporated valrubicin during the preparation process (Fig. S1C). The stable zeta potentials of the Val-ILs (Fig. S1D) made them suitable for testing in biological samples. Transmission electron microscopy (TEM) further confirmed that Val-ILs-αCD19 had an average size of 114 ± 28 nm, with the lipid bilayer averaging 7.7 ± 3 nm in size (as shown in Figs. 1C and S1E).

With these detailed characterizations and promising results in hand, the study proceeded to the assessment of the efficacy of Val-ILs in order to advance a new therapeutic nanoparticle technology for the treatment of acute leukemia.

### Val-ILs-αCD19 induces death of CD19-expressing cells

The in vitro evaluation of the impact of Val-ILs on the viability of B-cell acute lymphoblastic leukemia-1 (BALL-1) cell lines yielded significant results. Utilizing the XTT cell proliferation assay, three days after a single treatment of BALL-1 cells, the inhibitory concentration (IC_50_) for Val-ILs-αCD19 was determined to be 1000 particles per cell (*P* < 0.0001, Fig. 1D). Notably, treatment with 2000 particles per cell, equivalent to ~2 µL of the Val-ILs preparation per mL of culture, resulted in a significant decrease in the viability of BALL-1 cells. This finding was further substantiated through flow cytometry analysis, which confirmed the effect on cell viability (*P* < 0.0001, Fig. 1E). Importantly, there was no effect on the T-ALL Jurkat cells, which lack CD19 expression and consequently did not bind to Val-ILs-αCD19 (Fig. S2A). Fluorescent immunostaining and microscopy revealed that within one hour of incubation in the in vitro culture media, Val-ILs-αCD19 efficiently targeted the cell surface of BALL-1 cells, while Jurkat cells remained untargeted (Fig. S2B). Furthermore, treatment with Val-ILs-αCD19 at 2000 particles per cell for three days led to ~95% cell death in BALL-1 cells (*P* < 0.0001, compared with Val-ILs-IgG, Fig. S2C) without affecting the viability of Jurkat cells. These results strongly suggest that Val-ILs-αCD19 specifically induced cell death in CD19-targeted B-cells. Importantly, we noted that the Val-ILs contained only a minimal amount of valrubicin, measured at 1.52 × 10^−8^ pmol per particle using HPLC–MS/MS. Consequently, the amount of valrubicin required when loaded into Val-ILs-αCD19 was 32.7-fold lower (~3 × 10^−2^ µM) than the amount needed to achieve a similar effect on cell viability (1 μM) when the drug was directly applied to the cells. This striking difference highlights that Val-ILs are a highly efficient approach to reducing the drug concentration necessary for effective treatment.

We further assessed the effectiveness of Val-ILs-αCD19 on human peripheral blood mononuclear cells (PBMC) after 48 h of exposure in vitro. Using flow cytometry, it was determined that Val-ILs-αCD19 induced the death of healthy B-lymphocytes expressing CD19 at a rate exceeding 95% (Fig. 1F). In contrast, other cell populations that were negative for CD19 expression, such as T4- or T8- lymphocytes, were not sensitive to Val-ILs-αCD19. It should be noted that Val-ILs-IgG had no effect on the B-lymphocytes expressing CD19. Thus, in PBMC isolated from blood cultured ex vivo, Val-ILs-αCD19 could specifically target and eliminate the population of cells expressing CD19, such as B-lymphocytes, among a diverse range of hematopoietic cells. We also investigated the impact of Val-ILs-αCD19 on primary pediatric B-ALL samples isolated from the bone marrow (BM) of patients at diagnosis. The majority of living cells in these samples were CD19^+^ B-ALL cells. While Val-ILs-IgG had no effect, treatment with Val-ILs-αCD19 for 48 h eliminated 94 ± 6% of the B-ALL cells (*P* < 0.0001, Fig. 1G). This indicates that Val-ILs-αCD19 effectively eradicated the population of CD19^+^ B-ALL cells in primary B-ALL samples isolated from BM at the time of diagnosis.

These results demonstrate the potential clinical significance of Val-ILs-αCD19 in targeting and eliminating malignant B-ALL cells in pediatric patients.

### Val-ILs-αCD19 targets B-ALL cells in the BM and spleen of PDX mice

We used a mouse model to assess the in vivo effect of Val-ILs-αCD19 on primary pediatric B-ALL cells. This was achieved by transplanting the primary pediatric B-ALL cells into immunodeficient NSG mice, leading to the generation of two B-ALL PDX models referred to as #1 and #2. Thirty days after injection, ~80% of the BM cells recovered from the mice were B-ALL cells (hCD45^+^ hCD19^+^), while the remaining 20% of cells (hCD45^−^ hCD19^−^) consisted of endogenous murine cells present in the BM microenvironment, in which the leukemia cells were engrafted and had expanded. Before the in vivo experiments, we had to ensure that Val-ILs-αCD19 specifically targeted hCD19^+^ B-ALL cells. We, therefore, used trackable Val-ILs labeled with a green fluorescent dye (PKH67) during the preparation. This labeling allowed us to verify the specific binding of Val-ILs-αCD19 to human B-ALL cells and not to murine BM cells 18 h after treatment (Fig. S3A). We also assessed apoptosis using Annexin-V staining and observed that Val-ILs-αCD19 induced apoptosis in human B-ALL cells (*P* < 0.0001) while having no significant effect on murine BM cells (Fig. S3B). Furthermore, treatment with 2000 particles per cell affected the viability of 100% of B-ALL cells in PDX #1 and 95% in PDX #2 (Fig. S3C). An additional experiment involved growing green fluorescent protein-positive (GFP^+^) B-ALL cells on MS-5 stromal mesenchymal cells. Treatment with Val-ILs-αCD19 showed a significant impact on the viability of GFP^+^ cells within 24 h, while the supportive MS-5 cells continued to grow (Fig. 1H). These findings support the hypothesis that Val-ILs-αCD19 can effectively target leukemia cells while preserving mesenchymal cells, which play a supportive role for hematopoietic cells in the BM microenvironment.

After demonstrating that Val-ILs-αCD19 caused specific B-ALL cell death ex vivo, the study progressed to an assessment of whether treatment with Val-ILs-αCD19 might impact the in vivo development of B-ALL in PDX mice.

### Val-ILs-αCD19 affects the development of B-ALL in PDX mice

**Fig. 2:**
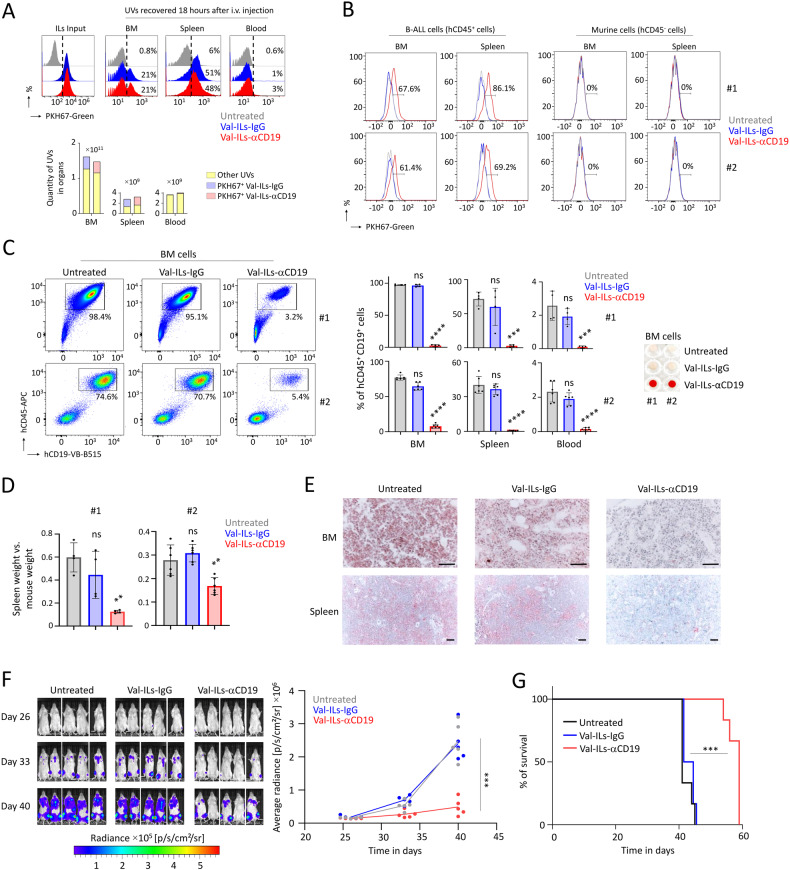
Treatment of PDX mice with Val-ILs-αCD19 affects the in vivo development of B-ALL. **A** Following the i.v. injection of trackable Val-ILs labeled with PKH67-Green in NSG mice, UVs were isolated from different organs 18 h later to measure PKH67-Green fluorescence by flow cytometry. Data showing that Val-ILs reached BM and spleen following i.v. injections. **B** Val-ILs-αCD19 targets B-ALL cells in the BM and spleen of PDX mice. Val-ILs labeled with PKH67-Green were injected at 10^11^ particles 15 days following the transplantation of 10^5^ B-ALL cells. Eighteen hours later, fluorescent Val-ILs binding, on hCD45^+^ B-ALL cells, as well as endogenous murine cells, were measured by flow cytometry in BM and spleen of PDX #1 and #2. **C** Mice were treated with three injections of 10^11^ Val-ILs on days 15, 20, and 25 post-transplantation, *n* = 4 mice per group for PDX #1 and *n* = 6 mice per group for PDX #2. Percentage of B-ALL cells in BM, spleen and blood measured by flow cytometry on day 35. Examples of cytometry dataset on BM and statistics for all organs. On the right is a picture of BM cell pellets on a 96-well plate, representative of PDX #1 and #2, showing that BM cells remained red-colored following treatment with Val-ILs-αCD19. **D** Data showing reduced splenomegaly in mice treated with Val-ILs-αCD19. The spleen weight was assessed in relation to the body weight of the mice. **E** Immunohistochemistry, following staining with hCD19 on BM and spleen sections, showing a decrease in the number of B-ALL cells (brown cells) in mice treated with Val-ILs-αCD19. Counterstaining was obtained by Giemsa staining. Data are representative of PDX #1 and #2, magnification ×40 for BM and ×20 for spleen, black scale bars represent 50 µm. **F** Mice were transplanted with bioluminescent B-ALL cells (PDX #1) and treated with 10^11^ Val-ILs on days 15, 20 and 25 post-transplantation. Bioluminescence images were taken on days 26, 33, and 40 post-transplantation, *n* = 5 mice per group. Average bioluminescence radiance over time is also shown on the right panel. **G** Kaplan–Meier survival plots showing that mice treated with Val-ILs-αCD19 survived longer than untreated mice or mice treated with Val-ILs-IgG, *n* = 6 mice per group. In this figure, data are shown as means ± SD. **C**, **D**, **F** *P* value calculated against untreated condition and measured by one-way ANOVA with Tukey’s multiple comparison tests; **P < 0.01; ***P < 0.001; ****P < 0.0001; ns, non-significant, **G** *P* value calculated against untreated condition and measured by Log-Rank (Mantel-Cox) test; ***P < 0.001.

We first aimed to characterize the distribution of Val-ILs in BM and spleen following injection into NSG mice without prior transplantation of B-ALL cells. Ensuring that these Val-ILs were not captured by B-ALL cells was crucial. We administered Val-ILs intravenously (i.v.) through the tail vein, and, considering previous findings demonstrating the arrival of ILs in organs in vivo a few hours after i.v. injection [49, 50], we chose to evaluate the binding of Val-ILs after an 18-h period. Tissues were crushed and UVs were obtained through precipitation after removing cells and debris. Using flow cytometry, the presence of Val-ILs labeled with PKH67-Green was detected in the BM (20% of UVs) and spleen (50% of UVs) but not in peripheral blood (Fig. 2A).

Subsequently, we aimed to evaluate the effective binding of Val-ILs-αCD19 to B-ALL cells by transplanting 10^5^ leukemia cells into NSG mice. Fifteen days after this transplantation, Val-ILs labeled with PKH67-Green were administered via i.v. injection. After 18 h, flow cytometry revealed the binding of Val-ILs labeled with PKH67-Green on over 60% of hCD45^+^ B-ALL cells in both the BM and spleen for both B-ALL PDX models (Fig. 2B). Notably, Val-ILs-IgG did not bind to the leukemia cells, and endogenous murine cells (hCD45^-^ cells) did not bind to the fluorescent Val-ILs-αCD19.

We further assessed how treatment with Val-ILs-αCD19 might affect the in vivo development of B-ALL in PDX mice. Animals received three injections of 10^11^ Val-ILs on days 15, 20, and 25 post-transplantation. On day 35, when leukemia had reached an endpoint in the control group, mice were sacrificed to recover various tissues. Flow cytometry revealed that Val-ILs-αCD19 induced significant death in over 90% of B-ALL cells in BM (*P* < 0.0001), spleen (*P* < 0.001) and blood (*P* < 0.001), compared to untreated mice or mice treated with Val-ILs-IgG (Fig. 2C). Moreover, BM in the treated mice was red, suggesting the presence of red blood cells, in contrast to untreated mice or mice injected with Val-ILs-IgG, whose BM cells were white, characteristic of B-ALL development. Val-ILs-αCD19 treatment also significantly attenuated splenomegaly (*P* < 0.01, Fig. 2D and Fig. S4). Immunohistochemistry in BM and spleen sections stained with an hCD19 antibody revealed that B-ALL cells in untreated mice or mice treated with Val-ILs-IgG had expanded considerably more than in mice treated with Val-ILs-αCD19 (Fig. 2E).

We next utilized lentiviral infection to create bioluminescent B-ALL cells expressing luciferase, which were then transplanted into mice. Three groups of mice were established. One group received treatment with Val-ILs-αCD19, and the other two groups received treatment with Val-ILs-IgG or physiological saline solution. The treatment was administered on days 15, 20, and 25 after transplanting 10^5^ bioluminescent B-ALL cells. Subsequently, luciferin was injected into the mice while they were asleep, allowing for the monitoring of the location of leukemia cells in living animals. We observed reduced bioluminescence in the group of mice treated with Val-ILs-αCD19 (five-fold decrease, *P* < 0.001, Fig. 2F). PDX mice treated with Val-ILs-αCD19 also exhibited extended survival compared to untreated PDX mice or mice treated with Val-ILs-IgG (*P* < 0.001, Fig. 2G).

These findings indicated that Val-ILs-αCD19 loaded with valrubicin had a substantial impact on the in vivo development of pediatric B-ALL in PDX mice, leading to improved survival rates.

### Val-ILs-αCD19 eradicates malignant B-ALL cells among CD34^+^ hematopoietic stem cells (HSC)

**Fig. 3:**
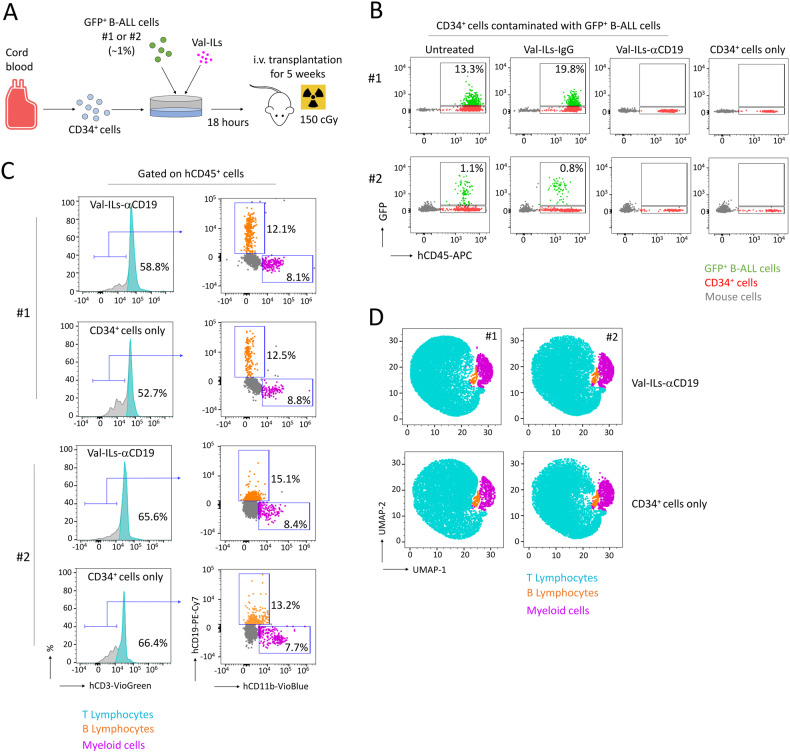
Val-ILs-αCD19 induces death of malignant B-ALL cells among CD34^+^ HSC. **A** Description of the procedure to deliberately contaminate CD34^+^ HSC isolated from human cord blood with ~1% of GFP^+^ B-ALL cells (isolated from PDX #1 or #2). **B** After 18 h of in vitro treatment with Val-ILs-IgG or Val-ILs-αCD19 (2000 particles/cell), cells were i.v. injected in NSG mice. Five weeks after the transplantation, GFP^+^ B-ALL cells were evaluated in BM by flow cytometry, as well as the reconstitution mediated by CD34^+^ HSC. Data showing how Val-ILs-αCD19 purged the malignant B-ALL cells among CD34^+^ HSC in vitro, while no leukemia GFP^+^ cells (green dots) were detected in the BM of transplanted mice. Data showing that treatment with Val-ILs-αCD19 did not affect the human hematopoietic reconstitution mediated by CD34^+^ HSC and characterized by hCD45^+^ expression (red dots). Flow cytometry gating (**C**) and UMAP data frames (**D**) performed on peripheral blood to assess the human reconstitution capacity of CD34^+^ HSC. Data gated on hCD45^+^ and GFP negative cells. Treatment with Val-ILs-αCD19 maintained the reconstitution capacity of CD34^+^ HSC, in all the hematopoietic lineages, compared with the uncontaminated control CD34^+^ cells (CD34^+^ cells only).

To address the risk of graft contamination with acute leukemia cells when using autologous peripheral blood stem cells (PBSC) for transplantation [51], we investigated the efficiency of Val-ILs in eradicating leukemia cells. This is particularly crucial for securing transplantation, and CD34^+^ tissue purging can be an effective method for eliminating malignant cells from the graft [51]. B-ALL cells from PDX #1 and #2 were transduced with a lentivirus to express a green fluorescent protein (GFP). We tested the ability of Val-ILs to eradicate cancer cells when CD34^+^ hematopoietic stem cells (HSC) isolated from human cord blood were deliberately contaminated with ~1% of GFP^+^ B-ALL cells. After 48 h of treatment with Val-ILs-αCD19 at a concentration of 2000 particles per cell, the B-ALL GFP^+^ cells were completely eliminated, while the CD34^+^ cells remained unaffected (Fig. S5). This finding demonstrates that a small amount of Val-ILs (~0.4 µL diluted in one mL of cell culture) was sufficient to eradicate all malignant B-ALL cells, leaving the CD34^+^ HSC unharmed. We further tested the effectiveness of Val-ILs-αCD19 in eradicating cancer cells when CD34^+^ HSC were contaminated with GFP^+^ B-ALL cells and then transplanted into NSG mice (Fig. 3A). Finally, no GFP^+^ B-ALL cells were detected in the BM, 5 weeks after transplantation, indicating that Val-ILs-αCD19 had successfully eradicated the leukemia cells (Fig. 3B). Additionally, the treatment did not impair the ability of CD34^+^ HSC to reconstitute human hematopoiesis across different lineages (Fig. 3C, D).

In summary, these results suggest that Val-ILs-αCD19 could be a valuable tool for removing leukemia cells that may contaminate autologous PBSC transplantation, enhancing the safety of the transplantation procedure.

### Development of Val-ILs loaded with αCD7 or αCD33 for T-ALL and AML therapies

**Fig. 4:**
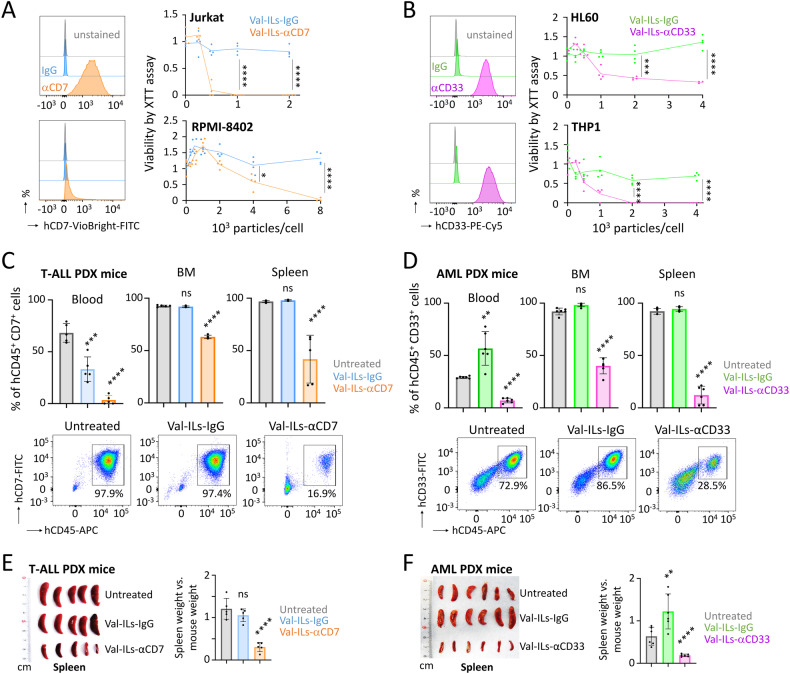
Development of Val-ILs loaded with αCD7 or αCD33 for T-ALL and AML therapies. **A** Flow cytometry plot showing the high expression of CD7 on Jurkat cells, and the low expression on RPMI-8402 cells. Treatment of these T-ALL cell lines with Val-ILs-αCD7 affects viability after three days, means of *n* = 4 biological replicates. **B** Flow cytometry plot showing CD33 expression on HL60 and THP1 cells. Treatment of these AML cell lines with Val-ILs-αCD33 affects viability after three days, means of *n* = 4 biological replicates. **C** T-ALL PDX mice were treated with three injections of 10^11^ Val-ILs on days 20, 25, and 30 post-transplantation data, which are shown as means ± SD, *n* = 5 mice per group. On day 45, the percentage of T-ALL cells (hCD45^+^ hCD7^+^) was measured by flow cytometry in the spleen, BM, and blood. Examples of cytometry dataset and statistics. **D** AML PDX mice were treated with three injections of 10^11^ Val-ILs on days 15, 20 and 25 post-transplantation, data are shown as means ± SD, *n* = 6 mice per group. On day 35, the percentage of AML cells (hCD45^+^ hCD33^+^) was measured by flow cytometry in the spleen, BM, and blood. Examples of cytometry dataset and statistics. Splenomegaly was reduced for T-ALL PDX mice treated with Val-ILs-αCD7 (**E**), as well as for AML PDX mice treated with Val-ILs-αCD33 (**F**). Pictures of spleens and statistics. The spleen weight was assessed in relation to the body weight of the mice. In this figure, **A**, **B** *P* value measured by two-tailed unpaired Student’s *t*-test; *P < 0.05; ***P < 0.001; ****P < 0.0001. **C**–**F** *P* value calculated against the untreated condition and measured by one-way ANOVA with Tukey’s multiple comparison test; **P < 0.01; ***P < 0.001; ****P < 0.0001; ns, non-significant.

We explored the potential of Val-ILs targeted with an antibody against CD7 on T-cell acute lymphoblastic leukemia (T-ALL) cell lines. Val-ILs-αCD7 effectively induced cell death in Jurkat cells expressing high levels of CD7, with a significant impact on cell viability achieved at a dose of 1000 particles per cell (100% decrease, *P* < 0.0001). Additionally, even RPMI-8402 cells with low CD7 expression had their viability affected by Val-ILs-αCD7 at a dose of 8000 particles per cell (98% decrease, *P* < 0.0001, Fig. 4A). Val-ILs loaded with an antibody against CD33, at a dose of 4000 particles per cell, also demonstrated efficient alterations in the viability of two acute myeloid leukemia (AML) cell lines, HL60 (68% decrease, *P* < 0.0001) and THP1 (100% decrease, *P* < 0.0001, Fig. 4B).

To further investigate the impact of these Val-ILs on in vivo T-ALL and AML development, pediatric T-ALL cells and AML cells were transplanted into NSG mice. Subsequent treatment with Val-ILs-αCD7 or Val-ILs-αCD33 involved three injections of 10^11^ Val-ILs. Flow cytometry revealed a notable decrease in leukemia cells in the BM, spleen, and blood when compared to untreated mice or mice treated with Val-ILs-IgG. It was observed that Val-ILs-αCD7 effectively induced cell death in T-ALL PDX mice, with a significant impact on cell viability (40% decrease in BM, *P* < 0.0001, Fig. 4C). Moreover, Val-ILs-αCD33 exhibited a pronounced ability to induce death of AML cells in PDX mice, resulting in a significant reduction in cell viability (60% decrease in BM, *P* < 0.0001, Fig. 4D). The results showed that treatment with Val-ILs significantly reduced splenomegaly in both T-ALL (*P* < 0.0001, Fig. 4E) and AML (*P* < 0.0001, Fig. 4F) PDX mice.

Overall, this experiment demonstrated the versatility of Val-ILs in targeting and inducing cell death in specific leukemia subtypes by incorporating different antibodies, such as anti-CD7 for T-ALL and anti-CD33 for AML.

### Development of Val-ILs targeting immune repressive cells for lymphoma immunotherapy

**Fig. 5:**
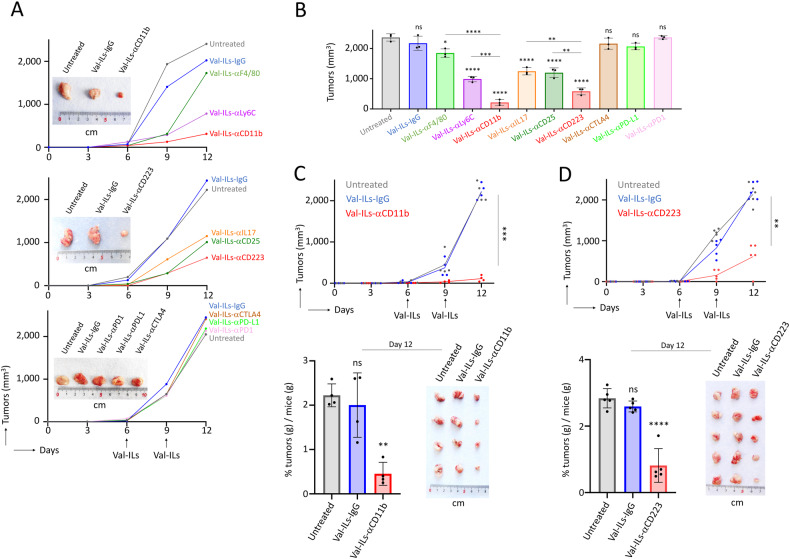
Val-ILs targets immunosuppressive cells for the immunotherapy of lymphoma. **A** C57BL/6 mice were subcutaneously injected with 10^6^ EL4 cells, on the body side. When tumors reached 50 mm^3^, mice were treated with Val-ILs i.v. injected into the tail vein on days 6 and 9 (arrows) at a dose of 10^11^ particles per injection. Different Val-ILs loaded with further antibodies were tested in order to target different populations of immune cells. Tumor growth volumes were assessed over time. Data showing that Val-ILs-αCD11b and Val-ILs-αCD223 efficiently attenuated tumor development in mice. Pictures of some tumors extracted from mice on day 12 are shown. **B** Tumor volumes were measured on day 12, with all the tested Val-ILs, *n* = 3 mice per group. **C** Val-ILs-αCD11b efficiently affect the tumor volume over time, means of *n* = 4 mice per group. On the bottom panel, pictures of the tumors and weights measured on day 12, *n* = 4 mice per group. **D** Val-ILs-αCD223 significantly affects the tumor volume over time, means of *n* = 5 mice per group. On the bottom panel, pictures of the tumors and weights measured on day 12, *n* = 5 mice per group. In this figure, data are shown as means ± SD, *P* values were calculated against untreated controls and measured by one-way ANOVA with Tukey’s multiple comparison test; *P < 0.05; **P < 0.01; ***P < 0.001; ****P < 0.0001; ns, non-significant.

While Val-ILs-αCD19 had a significant impact on the viability of Raji lymphoma cells in vitro (Fig. S6A), we found that fluorescent Val-ILs-αCD19 had more difficulty reaching the lymphoma tumor in vivo (Fig. S6B). Consequently, the three i.v. injections of Val-ILs-αCD19 at days 15, 20, and 25 did not alter the development of the lymphoma tumor (Fig. S6C, D, E). To address this issue, we explored the development of nanoparticles designed to target and induce cell death in immune cells involved in cancer immunosuppression. A lymphoma mouse model was established using immunocompetent C57BL/6 mice subcutaneously injected with EL4 cells. Various Val-ILs loaded with different antibodies were prepared to target MDSC [52–54], T4 regulatory (Treg) [55, 56] and T helper 17 (Th17) [56] lymphocytes, as well as the immune checkpoint molecules Programmed death-ligand 1 (PD-L1) or Programmed cell Death protein 1 (PD1) [57]. Screening these Val-ILs following two i.v. injections allowed the identification of two nanoparticles loaded with αCD11b or αCD223 antibodies as the most efficient treatment in vivo (Figs. 5A, B and S7A). When we repeated the experiment, Val-ILs-αCD11b, which targets myeloid cells, efficiently attenuated the development of lymphoma tumors in vivo (20-fold decrease, *P* < 0.001, Fig. 5C). In addition, Val-ILs-αCD223, which target T4 regulatory lymphocytes expressing CD223 [58, 59], were also found to effectively alter lymphoma growth in vivo (3-fold decrease, *P* < 0.01, Fig. 5D).

In conclusion, the study revealed that while Val-ILs might not directly reach the lymphoma tumor site, it is possible to develop nanoparticles for immunotherapy by specifically targeting immune suppressive cells within the tumor microenvironment, which can contribute to combating cancer growth.

### Val-ILs-αCD11b targets MDSC for lymphoma immunotherapy

**Fig. 6:**
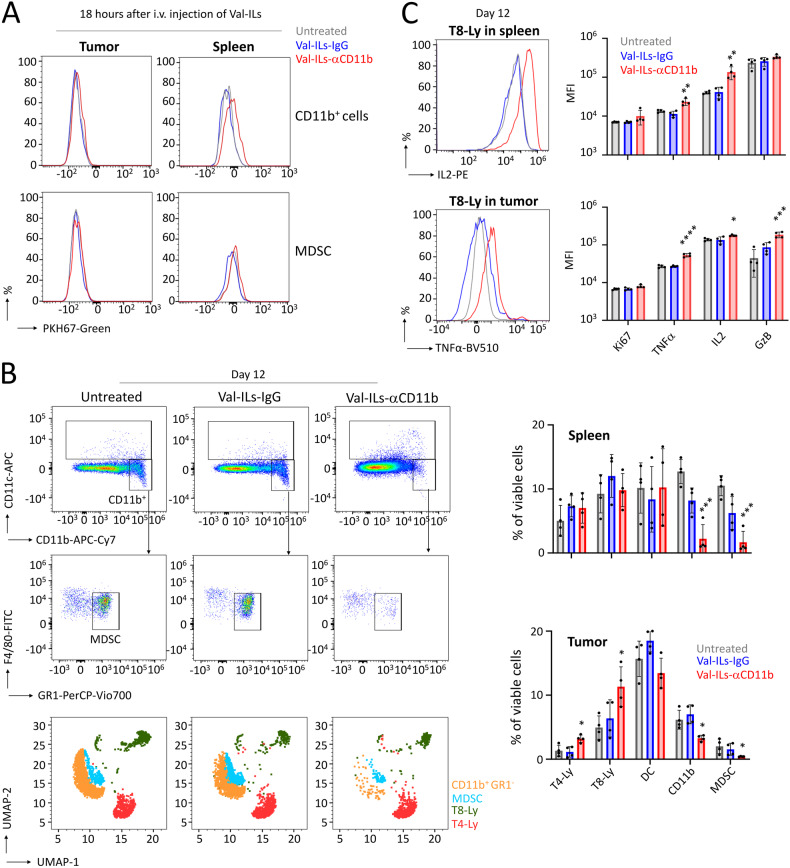
Val-ILs-αCD11b targets MDSC, leading to the activation of T8-Ly. **A** Six days following transplantation of EL4 cells, Val-ILs labeled with PKH67-Green were i.v. injected in mice at a dose of 10^11^ particles. Eighteen hours later, the binding of Val-ILs on CD11b^+^ cells and on MDSC immune cells was detected by flow cytometry in spleens but not in tumors. **B** Val-ILs-αCD11b were i.v. injected in mice at the dose of 10^11^ particles per injection on days 6 and 9. Example of flow cytometry dataset on immune cells on day 12, showing the reduction in myeloid-derived suppressor cells (MDSC) in the spleen of a mouse treated with Val-ILs-αCD11b. Flow cytometry UMAP data frame displaying a decrease in the number of MDSC (blue dots) in spleen. On the right are shown statistics of the immune system, in spleens and tumors, *n* = 4 mice per group. Dendritic cells (DC), T4 lymphocytes (T4-Ly), and T8 lymphocytes (T8-Ly). **C** Data showing the activation of T8-Ly measured by flow cytometry in spleens and tumors on day 12, following treatment with Val-ILs-αCD11b. Example of flow cytometry data set and statistics on the expression of markers involved in T8-Ly activation. Granzyme B (GzB), interleukin 2 (IL2), tumor necrosis factor-alpha (TNFα), and the proliferation marker Ki67, *n* = 4 mice per group. In this figure, data are shown as means ± SD, *P* values were calculated against untreated controls and measured by one-way ANOVA with Tukey’s multiple comparison test; *P < 0.05; **P < 0.01; ***P < 0.001; ****P < 0.0001. No statistic is shown when the *P* value is non-significant.

We demonstrated that Val-ILs-αCD11b efficiently targeted CD11b^+^ cells, including MDSC, within the spleen 18 h after i.v. injection, but not in EL4 lymphoma (Fig. 6A). Following two i.v. injections of 10^11^ particles at days 6 and 9, various immune cell populations were characterized using flow cytometry after the mice were sacrificed at day 12. The results revealed a reduced presence of MDSC in the spleens of mice treated with Val-ILs-αCD11b (6-fold decrease, *P* < 0.001), as well as in tumors (2-fold decrease, *P* < 0.05, Fig. 6B). Additionally, T8 lymphocytes within tumors were found to be more activated, as indicated by increased expression of granzyme B (GzB, *P* < 0.001), interleukin 2 (IL2, *P* < 0.05), and tumor necrosis factor-alpha (TNFα, *P* < 0.0001, Fig. 6C).

This experiment showed that Val-ILs-αCD11b precisely targeted MDSC, effectively inducing the death of this population of immunosuppressive cells. This, in turn, led to the further activation of T8 lymphocytes, optimizing the anti-cancer immune response in mice developing EL4 lymphoma.

### Val-ILs-αCD223 targets T4 regulatory lymphocytes for lymphoma immunotherapy

**Fig. 7:**
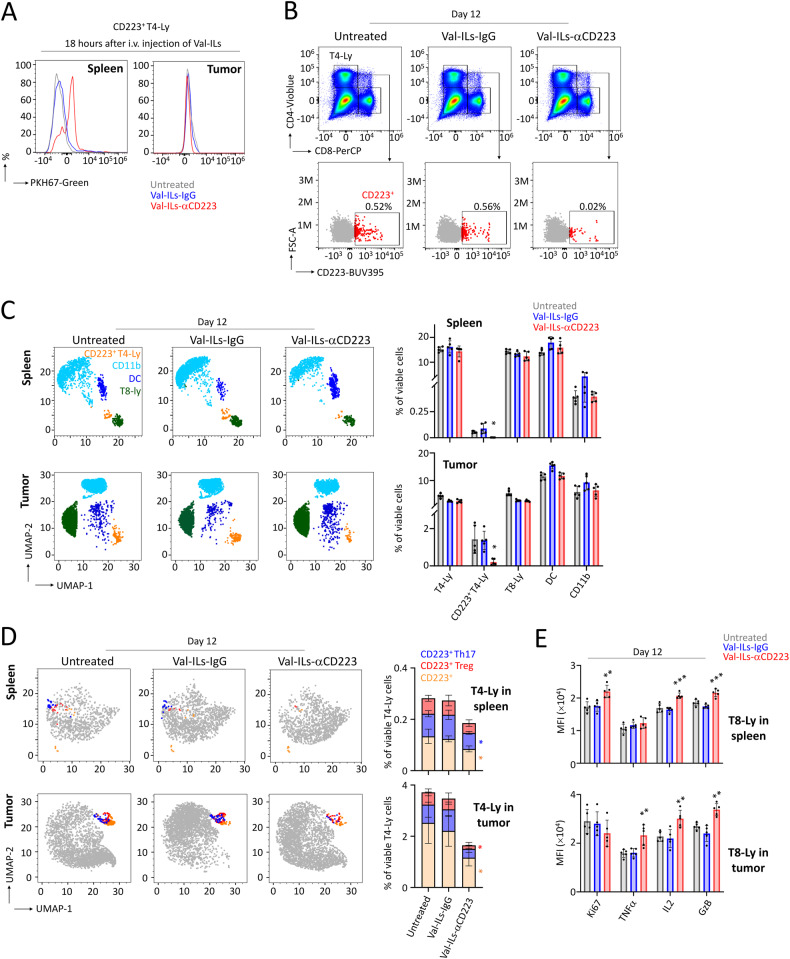
Val-ILs-αCD223 targets CD223^+^ T4-Ly, leading to the activation of T8-Ly. **A** Six days following transplantation of EL4 cells, Val-ILs labeled with PKH67-Green were i.v. injected in mice at a dose of 10^11^ particles per injection. Then, 18 h later, green fluorescent Val-ILs-αCD223 were detected by flow cytometry, showing binding on CD223^+^ T4-Ly in the spleen but not in the tumor. **B** Val-ILs-αCD223 were i.v. injected in mice, at a dose of 10^11^ particles, on days 6 and 9. Example of flow cytometry dataset on immune cells isolated from spleens on day 12, showing the reduction of CD223^+^ T4-Ly in the spleen of a mouse treated with Val-ILs-αCD223. **C** Flow cytometry UMAP data frame showing a decrease in CD223^+^ T4-Ly (orange dots) in spleens and tumors following treatment with Val-ILs-αCD223. The other populations of immune cells were not affected, such as DC (dark blue dots), T8-Ly (green dots), or myeloid CD11b^+^ cells (clear blue dots). Statistics of the immune system analyzed by UMAP in spleens and tumors, *n* = 5 mice per group. **D** Flow cytometry UMAP data frame showing decrease in T4-Ly regulatory cells expressing CD25 and FoxP3 (CD223^+^ Treg, red dots), Th17 T4-Ly expressing IL17 (CD223^+^ Th17, blue dots), as well as the remaining CD223^+^ T4-Ly (CD223^+^, orange dots), in spleens and tumors on day 12, following treatment with Val-ILs-αCD223. Other populations of immune cells are shown (gray dots). Statistics of the CD223^+^ T4-Ly populations analyzed by UMAP, in spleens and tumors, *n* = 5 mice per group. **E** Data showing the activation of T8-Ly measured by flow cytometry in spleens and tumors on day 12 following treatment with Val-ILs-αCD223. Example of flow cytometry data set and statistics on the expression of markers involved in T8-Ly activation. Granzyme B (GzB), interleukin 2 (IL2), tumor necrosis factor-alpha (TNFα), and the proliferation marker Ki67, *n* = 5 mice per group. On this figure, data are shown as means ± SD, *P* values were calculated against untreated controls and measured by one-way Anova with Tukey’s multiple comparison test; *P < 0.05; **P < 0.01; ***P < 0.001. No statistic is shown when the *P* value is non-significant.

We demonstrated that Val-ILs-αCD223 efficiently targeted CD223^+^ cells within the spleen but not within the EL4 tumor 18 h after i.v. injection (Fig. 7A). After two injections of 10^11^ particles on days 6 and 9, the populations of CD223^+^ T4 lymphocytes in both spleens and tumors were significantly affected by Val-ILs-αCD223 (Fig. 7B), with no specific effect on other immune cells (Fig. 7C). Further analysis revealed that Val-ILs-αCD223 effectively reduced the population of cells expressing FoxP3 and CD25 in tumors (3-fold decrease of Treg cells, *P* < 0.05) and interleukin 17 (IL17) in spleens (3-fold decrease of Th17 cells, *P* < 0.05), as well as the population of CD223^+^ cells which were not Th17 or Treg cells in both spleens and tumors (2-fold decrease, *P* < 0.05, Fig. 7D). Moreover, there was a notable increase in the activation of T8 lymphocytes within the tumors, as indicated by elevated expression of several markers detected by flow cytometry, including GzB (*P* < 0.01), IL2 (*P* < 0.01), and TNFα (*P* < 0.01, Fig. 7E).

In summary, Val-ILs-αCD223 effectively reduced the number of T4 lymphocytes, including Treg and Th17 immunosuppressive cells, leading to a significant activation of T8 lymphocytes. This suggests the relevance of Val-ILs-αCD223 for immunotherapy in mice developing EL4 lymphoma.

### Combination of Val-ILs targeting distinct populations of immunosuppressive cells increases the survival of lymphoma mice

**Fig. 8:**
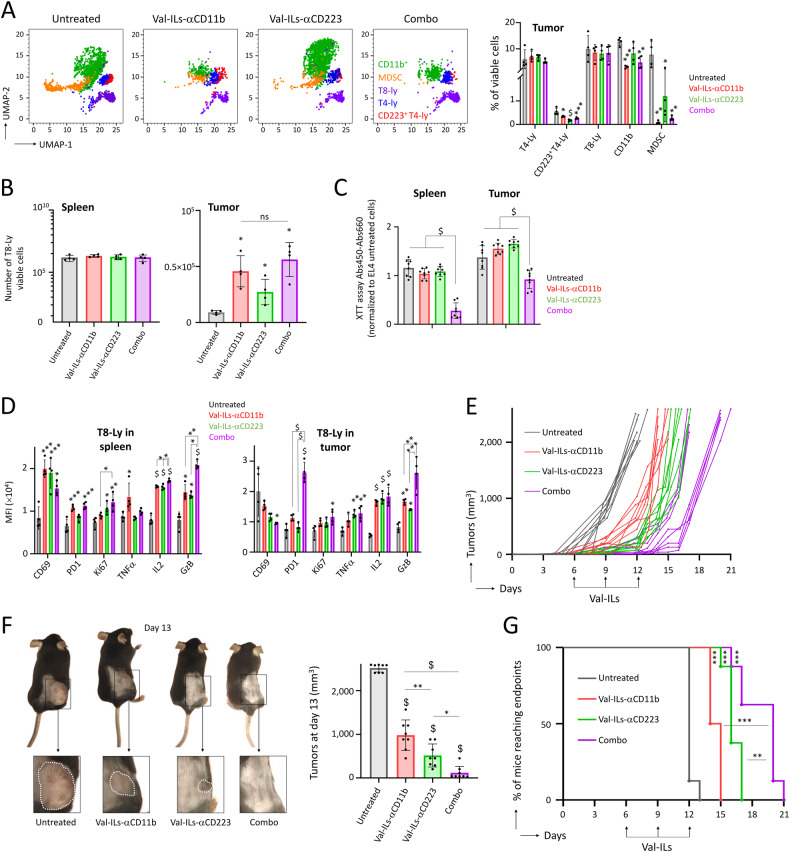
Combo Val-ILs reduces lymphoma development in vivo. C57BL/6 mice were subcutaneously injected with 10^6^ EL4 cells on the body side. When tumors reached 50 mm^3^, mice were treated with Val-ILs-αCD11b, Val-ILs-αCD223, or a combination of Val-ILs-αCD11b and Val-ILs-αCD223 (Combo), at a dose of 2 × 10^11^ particles, on days 6 and 9. **A** Flow cytometry UMAP data frame and statistics showing that treatment with Combo Val-ILs affected both MDSC (orange dots) and CD223^+^ T4-Ly (red dots) in tumors on day 12, *n* = 4 mice per group. **B** On day 12, T8 lymphocytes were isolated and quantified from spleens and tumors. Data show that treatment of mice with Combo Val-ILs does not increase the quantity of T8 lymphocytes in tumors, *n* = 4 mice per group. **C** On day 12, T8 lymphocytes were isolated from spleens and tumors and co-cultured with EL4 cells at a ratio of 1:100, and viability was assessed by XTT assay after 48 h of treatment, *n* = 8 biological replicates per group. Data show that T8 lymphocytes isolated from mice treated with Combo Val-ILs affect the viability of EL4 cells more efficiently. **D** Flow cytometry data showing that treatment with Combo Val-ILs activate the expression of PD1 (*P* < 0.0001) and GzB (*P* < 0.01) on T8 lymphocytes, *n* = 4 mice per group. **E** Mice were treated with Val-ILs-αCD11b, Val-ILs-αCD223, or a combination of Val-ILs-αCD11b and Val-ILs-αCD223 (Combo). Val-ILs were i.v. injected into the tail vein on days 6, 9 and 12 (arrows), at a dose of 2 × 10^11^ particles per injection. Tumor growth volumes were assessed over time, *n* = 8 mice per group. **F** Pictures of mice on day 13, with outlined tumors (ached white lines) and statistics on tumor volume, *n* = 8 mice per group. **G** Kaplan–Meier survival plots showing that mice treated with Val-ILs-αCD11b or Val-ILs-αCD223 survived longer than untreated mice, and the survival was even better when mice were treated with Combo Val-ILs, *n* = 8 mice per group. In this figure, **A**–**D**, **F** data are shown as means ± SD, *P* values were measured by one-way ANOVA with Tukey’s multiple comparison test; *P < 0.05; **P < 0.01; ***P < 0.001; ^$^*P* < 0.0001. No statistic is shown when the *P* value is non-significant. **G** *P* value measured by Log-Rank (Mantel–Cox) test; **P < 0.01; ***P < 0.001.

Anti-CD11b and anti-CD223 antibodies had no antagonistic effect, and there was no impaired viability after ex vivo treatment (Fig. S7B). Furthermore, Val-ILs-αCD11b and Val-ILs-αCD223 did not target EL4 cells (Fig. S7C). We investigated the combinatorial effect mediated by treatment with Val-ILs-αCD11b and Val-ILs-αCD223 on immune cells isolated from the spleen of mice six days after the subcutaneous injection of EL4 lymphoma cells. Nanoparticles were produced, loaded with both antibodies and administered to immune cells ex vivo. When Val-ILs were loaded with both antibodies, their ability to affect the viability of T4 lymphocytes expressing CD223 was lost (Fig. S7D). Therefore, it was preferable to treat mice with a combined administration of Val-ILs-αCD11b and Val-ILs-αCD223 (Combo Val-ILs) rather than Val-ILs loaded with both antibodies. Flow cytometry showed that a treatment with Combo Val-ILs efficiently reduced both MDSC and CD223^+^ T4 lymphocytes in tumors (Fig. 8A). Combo Val-ILs did not further increase the amount of T8 lymphocytes detected in the tumors compared with Val-ILs-αCD11b or Val-ILs-αCD223 alone (Fig. 8B). However, on day 12, T8 lymphocytes isolated ex vivo from the tumors and spleens of mice treated with Combo Val-ILs were significantly more effective in altering EL4 cell viability in vitro compared with T8 lymphocytes isolated from mice treated with either Val-ILs-αCD11b or Val-ILs-αCD223 alone (*P* < 0.0001, Fig. 8C). This was accompanied by a marked increase in the expression of PD1 and GzB in T8 lymphocytes, indicating their activation (Fig. 8D). Treatment with Combo Val-ILs was shown to significantly reduce tumor volumes compared to treatments with Val-ILs-αCD11b or Val-ILs-αCD223 alone (Fig. 8E, F). The survival of mice treated with Combo Val-ILs was also significantly longer (Fig. 8G), emphasizing the potential of these nanoparticles for immunotherapy in lymphoma.

In summary, the study demonstrated that a combination of Val-ILs targeting and inducing death of distinct populations of immunosuppressive cells could lead to increased survival in lymphoma mice.

## Discussion

The use of ILs represents an innovative therapeutic approach that has been the subject of active research in preclinical models of solid cancer, showing promising results [16]. Our study aimed to develop antigen-targeting ILs loaded with valrubicin, a hydrophobic analog derivative of doxorubicin, which is an anthracycline. Valrubicin is a key component used for intravesical chemotherapy to treat carcinoma in situ of the bladder [41–45]. Our research revealed that valrubicin, known for its lipophilic properties, is readily integrated into the lipid layer of Val-lLs. Preclinical studies conducted on various murine models have shown that this novel nanoparticle exhibits high efficacy in the treatment of hematological malignancies. Up to this point, all the CD19-targeting ILs designed for other B-cell malignancies have utilized hydrophilic drugs, including vincristine, doxorubicin, imatinib, or rapamycin, encapsulated within the internal aqueous compartment [31–35]. Patients diagnosed with B-ALL typically undergo multi-agent chemotherapy, such as vincristine and dexamethasone, as part of their treatment regimen [1–4]. However, when we created Val-ILs-αCD19 loaded with these two hydrophilic compounds, we noticed that the combined efficacy of these drugs in inducing B-ALL cell death was significantly compromised once they were encapsulated within ILs, as shown in Fig. S8. These findings suggest that ILs containing hydrophilic drugs were less effective than ILs incorporating the lipophilic prodrug valrubicin. This disparity may be attributed to the ease of incorporating a lipophilic drug into the lipid layer of ILs during nanoparticle production while maintaining the encapsulation of hydrophilic drugs appears to be a more challenging task.

In patients with B-ALL, chimeric antigen receptor T cell (CAR-T) immunotherapy has shown a 70% response rate, but a concerning 10–20% of pediatric responders experience relapse with epitope loss [60, 61]. Several mechanisms have been implicated in this phenomenon, including alternatively spliced *CD19* transcripts lacking the epitope targeted by CAR-T cells [60], *CD19* mutations that result in truncated proteins [61], and *CD19* epigenetic repression contributing to antigen escape [62]. Our study revealed the continued expression of CD19 on the cell surface of residual cells in PDX mice treated with Val-ILs-αCD19, as shown in Fig. S9A. Among the 23 B-ALL PDX mice treated with Val-ILs-αCD19, only one mouse failed to respond to the treatment, as indicated in Fig. S9B. Further investigation revealed that hypermethylation of the CD19 promoter, as previously observed [62], was the underlying cause of antigen-negative escape from Val-ILs-αCD19, but this phenomenon was observed exclusively in the non-responsive mouse, as presented in Fig. S9C.

In our preclinical study, the administration of Val-ILs did not have any noticeable impact on the weight of the mice over the course of the experiment, as demonstrated in Fig. S10A. Additionally, Val-ILs treatment did not result in significant toxicity in terms of blood parameters in the mice, except for a temporary increase in white blood cells, specifically monocytes and granulocytes, observed one day after the first i.v. injection. This effect was mitigated within a few days, despite two subsequent injections of Val-ILs, as depicted in Fig. S10B.

Just as ILs loaded with a prodrug offer unique advantages, antibody-drug conjugates (ADCs) combine the precision of monoclonal antibodies with the potency of highly cytotoxic agents, and there are several ADCs currently in use for treating hematological malignancies [63, 64]. Nonetheless, toxicity remains a significant concern [65]. While ILs employ a strategy similar to ADCs, they offer certain advantages by modifying the pharmacokinetic properties of drugs, facilitating faster intracellular penetration and higher drug concentrations within target cells [65, 66]. Numerous experiments have focused on enhancing the solubility, stability, circulation time, and drug-targeting properties of ILs [11–14]. The lipophilic nature of valrubicin amplifies its cytotoxic effects by elevating intracytoplasmic drug concentrations and enabling it to traverse cell membranes with lower systemic toxicity [44]. Our Val-ILs contained only a minimal amount of valrubicin, quantified at 1.52 × 10^−8^ pmol per particle using HPLC-MS/MS. Therefore, the utilization of Val-ILs loaded with valrubicin required 32.7-fold less of the drug to achieve a similar effect on cell viability compared to direct drug application. In our preclinical study involving PDX mice, we calculated that just 0.145 mg/kg of valrubicin, when incorporated into Val-ILs, was enough to reduce the expansion of leukemia cells in vivo. Val-ILs combine the precision of monoclonal antibodies with the potency of highly cytotoxic agents, potentially diminishing the severity of side effects by selectively delivering the drug to the tumor site. Traditionally, chemotherapy for leukemia involves frequent administration and is associated with severe side effects due to the non-specificity of the drugs. However, since Val-ILs contained a very low dose of the lipophilic drug and an antibody designed to specifically target the population of leukemia cells, the risk of in vivo side effects should be significantly reduced. One potential limitation of Val-ILs is that the particles loaded with antibodies targeting CD19, CD7, or CD33 might affect not only ALL or AML cells but also healthy hematopoietic cells in BM, including B- and T-lymphocytes, as well as myeloid cells. However, this limitation is present in other therapeutic strategies, including ADCs and CAR-T immunotherapies.

Another challenge detected in our preclinical study was the ability of Val-ILs to penetrate lymphoma tumors. In response, we developed Val-ILs targeting immunosuppressive cells in the spleen, including Val-ILs-αCD11b, which target MDSC, and Val-ILs-αCD223, which target lymphocyte-activation gene 3 (LAG-3 or CD223) on T4 lymphocytes. These nanoparticles significantly influenced the development of lymphoma in vivo. Furthermore, the combination therapy involving both Val-ILs-αCD11b and Val-ILs-αCD223 showed superior effectiveness in preventing the growth of lymphoma tumors. Interestingly, we found that treating mice with a combination of Val-ILs-αCD11b and Val-ILs-αCD223 was more beneficial than using Val-ILs loaded with both antibodies. One possible explanation for this observation could be that Val-ILs loaded with both antibodies might be captured by CD11b-expressing cells, which are more abundant among immune cells. Consequently, Val-ILs loaded with both antibodies may not effectively target the rarer population of cells, such as CD223-positive T4 lymphocytes, essential for lymphoma prevention.

In conclusion, our findings strongly indicate that immunotargeting with liposomes containing a lipophilic antitumor prodrug is a novel and effective strategy for targeting and inducing the death of leukemia cells. Furthermore, we have demonstrated that these nanoparticles serve as a valuable tool to target and induce the death of immunosuppressive cells, thus enhancing the antitumor immune response in the context of lymphoma. The interesting results obtained from our preclinical tests hold significant potential and should be further explored, serving as a stepping stone for future clinical trials in the field of hematology. This study is a promising preclinical demonstration of the effectiveness and ease of preparation of Val-ILs, which is a novel nanoparticle technology that could potentially yield effective therapies for hematological cancers based on targeted vesicle-mediated cell death.

## Materials and methods

### ILs loaded with valrubicin

L-α-phosphatidylcholine (P7443, Merck), cholesterol (C8667, Merck), DSPE-PEG-square (2000) (880136P, Merck) and DSPE-PEG-NHS (5000) (06030500706, SINOPEG) were prepared in chloroform (Merck) with a respective molar ratio of 100:37:1.5:0.2, to a final volume of 26 µl. To this mix, we added 26 nmol of valrubicin (SML2516, Merck) reconstituted in DMSO (Merck). Then, chloroform was evaporated with nitrogen, making a thin lipid film in a round-bottom flask by the removal of organic solvent. The lipid film was then reconstituted in 1 mL of filtrated PBS 1× preheated to 65 °C. Following 10 min of incubation at 65 °C, the mixture was vortexed for 1 min, then after stabilization for 30 min at 65 °C, the vesicles were sonicated for 5 cycles (30 s on-off) at an amplitude of 20% to form UVs that were stabilized at 65 °C for 30 min. UVs targeting B-ALL cells, T-ALL cells, or AML cells were then incubated with 2 µg of an antibody (Table S1). The reaction was stopped by adding 1 mM of glycine. In order to purify the Val-ILs, two consecutive dialyses (one of 2 h and another one overnight) were carried out in one liter of PBS 1×, with 300 KDA dialysis membranes (11550970, Thermo Fisher Scientific). To prove that the antibodies used to prepare Val-ILs have no cytotoxic effect on their own, anti-hCD19 (1:100), anti-hCD7 (1:100), or anti-hCD33 (1:100) antibodies were reciprocally tested on BALL1, Jurkat and THP1 cells, and viability was analyzed by XTT cell proliferation assay (Thermo Fisher Scientific) following 3 days of cell culture. The same protocol was followed to prepare Val-ILs used on mice developing the EL4 lymphoma. All antibodies used to prepare Val-ILs, including IgG isotypes control antibodies, are described in Table S2. For PKH67^+^ Val-ILs, 5 µL of PKH67 (MINI67-1KT, Merck) was added to lipids before sonication. After stabilization, purification was performed by ultracentrifugation at 120,000×*g* (Optima XE-90 ultracentrifuge, Beckman Coulter) for 90 min at 4 °C. Val-ILs pellets were suspended in filtrated PBS 1×, and they were incubated with 2 µg of antibodies for 2 h at room temperature under agitation. The reaction was stopped by adding 1 mM of glycine. To prepare ILs with vincristine (V8388, Merck) and dexamethasone (D1756, Merck), both compounds reconstituted in 1 mL of filtrated PBS 1× at 1 µM each, was directly applied on the dried lipid film, then vortexed and sonicated following the same procedure. The size and concentration of Val-ILs were characterized by nanoparticle tracking analysis (NTA) using a NanoSight NS300 Instrument (Malvern Instruments, Malvern, England). The Zeta potential of Val-ILs was measured by a Zetasizer Nano-ZS (Malvern Instruments, Malvern, England). Valrubicin dosage with colorimetry was performed at 560 nm (EnVision 2104, Perkin Elmer).

### Establishment of ALL and AML PDX models and treatments with Val-ILs

NOD/SCID/γc^−/−^ (NSG) mice (Charles River) were bred, raised, and housed under pathogen-free conditions. To induce leukemia, we i.v. injected 10^5^ B-ALL, 10^5^ AML, or 5 × 10^5^ T-ALL in a volume of 300 μL of physiological saline solution in the tail vein of non-irradiated 7–16-week-old male and female NSG mice. Males and females were randomly allocated to experimental groups, and no blinding method was used for injection. Following RTqPCR and CGH array on hCD45^+^ cells, isolated with magnetic beads, B-ALL PDX cells ≠1 displayed an *IGK::MYC* translocation, as well as microdeletions in *CDKN2A* and *RB1* genes, B-ALL cells ≠2 displayed a *TEL::AML1* chromosomal translocation, as well as microdeletions in *IGLL5*, *PIK3C3* and *RB1* genes. T-ALL PDX cells displayed a STIL (*SCL::TAL1* interrupting locus), as well as deletions in *LEF1* and *CDKN2A* genes. AML PDX cells displayed an *MLL::AF9* chromosomal translocation. Val-ILs were i.v. injected into the tail vein three times every 5 days (days 15, 20, and 25 for B-ALL and AML, and days 20, 25, and 30 for T-ALL) at a dose of 10^11^ particles in 300 µL of physiological saline solution. After tail vein blood sampling from PDX mice, white blood cells were recovered following hemolysis (NH4Cl 150 mM, KHCO3 10 mM, EDTA 0.1 mM, pH 7.4). Tibias and femurs from the two bottom legs were crushed in a mortar, in PBS 1×, and total BM cells were filtered with a sterile cell strainer (70 µm). Spleens were also crushed and filtered with the sterile cell strainer in hemolysis solution, and the cells were washed with PBS 1×. For flow cytometry analysis or immunohistochemistry, we recovered blood, spleen and BM cells at day 35 for B-ALL and AML and at day 45 for T-ALL, following previously described protocols [67]. We used rabbit anti-hCD19 (1:100, SAB5500047, Merck) for immunohistochemistry that was performed by the ImaFlow platform (Université de Bourgogne, Dijon, France) under an AxioScope microscope (Zeiss). Eighteen hours after the injection of PKH67^+^ Val-ILs in PDX mice, cells were recovered, by centrifugation at 500×*g*, from BM, spleen and blood to detect PKH67^+^ Val-ILs binding, by flow cytometry. To analyze the distribution, Val-ILs were i.v. injected in NSG mice, then 18 h later, cells were recovered by centrifugation at 500×*g* from BM, spleen and blood, debris were removed from the supernatant by centrifugation at 10,000×*g*. From the supernatant, UVs were isolated by pull-down, following previously described procedures [68] with a specific kit (15254394, Thermo Fisher Scientific), and analyzed by flow cytometry.

### Bioluminescence imaging

We created PDX models that developed stable bioluminescence by infecting B-ALL cells #2 with a lentivirus expressing both GFP and luciferase [67]. Then, we transplanted these cells into mice that were used later for bioluminescence imaging. The lentivirus was produced in HEK293 cells after transduction with Lipofectamin 2000 (Thermo Fisher Scientific) of the pCCLc-MNDU3-Luciferase-PGK-EGFP-WPRE vector (Addgene, #89608), as well as PAX2 (Addgene, #12260) and pCMV-VSV-G (Addgene, #8454) plasmids. After 2 days, viral supernatants were recovered, and 6-well plates were incubated for 4 h with retronectin (Takara, Ozyme). Viral supernatants were then spinoculated for 30 min at 4000×*g*. Cells were cultured on these plates for three days in StemMACS media (Miltenyi Biotech). Lentiviral transduced cells (GFP^+^) were sorted on a FACSAriaIII cell sorter (BD Biosciences) and transplanted in NSG mice to generate bioluminescent PDX. Following isoflurane-induced anesthesia, animals were imaged 15 min after d-luciferin (Merck) injection, at 150 mg/kg body weight, using an IVIS Lumina III system coupled with Living Image acquisition and analysis software version 4.0 (Perkin Elmer).

### Establishment of in vivo EL4 lymphoma models and treatments with Val-ILs

For the murine lymphoma model, EL4 cells (TIB-39, ATCC) were cultured in DMEM media (Dominique Dutscher) supplemented with 10% fetal bovine serum (FBS) (Dominique Dutscher) and Penicillin-Streptomycin-Amphotericin (PSA) (Pan Biotech). For the study in vivo, 10^6^ EL4 cells were subcutaneously injected in a volume of 100 μL of physiological saline solution on the body side of C57BL/6 mice (Envigo). For injection, males and females were shaved and randomly assigned to experimental groups, and no blinding method was used for injection. Mice were treated when tumors reached 50 mm^3^ (day 6). Val-ILs were i.v. injected into the tail vein at days 6 and 9 at a dose of 10^11^ particles in 300 µL of physiological saline solution. Tumor growth was measured over time. In order to perform flow cytometry on immune cells isolated from the spleen and tumor, mice were euthanized 12 days after the injection of EL4 cells when the tumor reached the endpoint, >2000 mm^3^, in the untreated control group. For the combinatorial study, Val-ILs were i.v. injected into the tail vein on days 6, 9, and 12 at a dose of 2 × 10^11^ particles for each condition. Spleens were crushed and filtered with the sterile cell strainer in hemolysis solution, and the cells were washed with PBS 1×. Tumors were chopped up into 3–4 mm pieces with a sterile scalpel. Tumor samples were then placed in 2.5 mL of the dissociation buffer containing 60 U/mL of Collagenase, Type 1 (CLS-I, LS004194, Cell Systems), 30 U/mL of Collagenase, Type 2 (CLS-II, LS004174, Cell Systems), 60 U/mL of Collagenase, Type 4 (CLS-IV, LS004186, Cell Systems), and 25 µg/mL of DNAse 1 (04536282001, Merck) in filtered PBS 1×. The mixture was incubated under agitation at 37 °C for 45 min. Cell suspension was filtered through 30 µm separation filters (130-041-407, Miltenyi Biotech) and centrifuged at 500 *g*. Mice were also i.v. injected with PKH67^+^ Val-ILs (10^11^ particles, at day 6) and sacrificed 18 h after to analyze, by flow cytometry, the presence of UVs in the tumor and spleen, as well as the binding of fluorescent Val-ILs on immune cells. The supernatant was centrifuged at 10,000×*g* to remove debris, and then UVs were isolated from the supernatant by pull-down with a specific kit (15254394, Thermo Fisher Scientific) and analyzed by flow cytometry.

### Activity of T8 lymphocytes isolated ex vivo on EL4 cells

C57BL/6 mice were injected with 10^6^ EL4 cells, and when tumors reached 50 mm^3^, mice were treated with Val-ILs. At day 12, T8 lymphocytes were recovered from tumors and spleens, with anti-CD8 magnetic beads (130-104-075, Miltenyi Biotec) and cultured in RPMI-1640 media supplemented with 10% FBS, 1% PSA, MEM 1× (11140035, Thermo Fisher Scientific), l-glutamine 2 mM (25030149, Thermo Fisher Scientific), HEPES 10 mM (15630080, Thermo Fisher Scientific), pyruvate sodium 1 mM (11360070, Thermo Fisher Scientific) and β-mercaptoethanol 5.5 nM (21985023, Thermo Fisher Scientific). T8 lymphocytes were enumerated and co-cultured with EL4 cells, at a ratio of 1:100. Viability was assessed by XTT cell proliferation assay (Thermo Fisher Scientific), following 48 h of treatment.

### Cell culture and treatment with Val-ILs

BALL-1 (DSMZ), Jurkat (ATCC), RPMI-8402 (ATCC), THP1 (ATCC), and HL60 (ATCC) cells were cultured in RPMI-1640 media (Dominique Deutscher) supplemented with 10% FBS and 1% PSA. For the ex vivo experiments, we used cells freshly isolated from the BM of PDX mice, cultured in StemMACS media (Miltenyi Biotec) supplemented with 1% PSA. Murine MS-5 mesenchymal stromal cells (ACC-441, DSMZ) were cultured in IMDM media (Thermo Fisher Scientific) with 10% FBS and 1% PSA. When cell confluence reached 80%, we administered GFP^+^ B-ALL cells in StemMACS media, and, following 5 h of binding, 6-well plates were treated with Val-ILs-αCD19. Multiple pictures per well were taken every 12 h using an IncuCyte S3 (Sartorius). For cell culture, cells were grown in an incubator at 37 °C in a humid atmosphere and 5% CO_2_ pressure. Viability was assessed by XTT cell proliferation assay, measured by absorbance at 450 nm minus absorbance at 660 nm (EnVision 2104, Perkin Elmer), following previously described procedures [67]. Cells were treated with different concentrations of Val-ILs, at a single dose treatment at the beginning of the experiment, and XTT cell proliferation assay was performed 72 h after the treatment. BALL-1 and Jurkat cells were treated for 2 h with 2000 particles/cell, and then we performed fluorescence microscopy, following previously described procedures [67].

### Human peripheral blood mononuclear cell (PBMC) isolation and Val-ILs treatment

PBMC were isolated following Pancoll (Pan Biotech) density gradient centrifugation. Viability, exceeding 90%, was assessed using Trypan Blue (Thermo Fisher Scientific) before the treatments, and cells were cultured in RPMI-1640 media with 10% FBS and 1% PSA. PBMC was divided into three wells and treated with 2000 Val-ILs/cell. Cell viability was analyzed by flow cytometry 48 h after the treatment.

### CD34^+^ cord blood isolation, contamination with B-ALL cells, treatment with Val-ILs and transplantation in NSG mice

Mononuclear cells were isolated from two cord blood samples following Pancoll (Pan Biotech) density gradient centrifugation. CD34^+^ cells were recovered with magnetic beads (130-046-702, Miltenyi Biotec) and divided into 4 wells in 1 mL of StemMACS media (Miltenyi Biotec), supplemented with PSA (Pan Biotech), human stem cell factor (SCF, 25 ng/mL, 130-093-991, Miltenyi Biotec), human Interleukin 3 (IL3, 10 ng/mL, 130-093-908, Miltenyi Biotec), and human Interleukin 6 (IL6, 10 ng/mL, 130-095-365, Miltenyi Biotec). CD34^+^ cells were deliberately contaminated with 1% of GFP^+^ B-ALL cells (#1 or #2). The mix of CD34^+^ and GFP^+^ B-ALL cells was treated with 2000 particles/cell. Eighteen hours later, cells were washed with PBS 1×, then 1.5 × 10^5^ cells in physiological saline solution were transplanted in NSG mice and irradiated 24 h before the transplantation at a sublethal dose of 1.5 Gray (BioMEP, Bretenière, France). After 5 weeks, the development of B-ALL was monitored by GFP expression in BM. CD34^+^ HSC reconstitutions were assessed by flow cytometry in PB and BM. We also determined the disappearance of GFP^+^ cells in vitro by flow cytometry 48 h after the treatment.

### Treatment of primary B-ALL samples with Val-ILs-αCD19

Cell isolated from the BM collected at diagnosis of patients with B-ALL were cultured in StemMACS medium with 1% PSA. The viability, exceeding 90%, was assessed using Trypan Blue before treatments. Cells were divided into three wells and treated with 2000 Val-ILs/cell. Cell viability was analyzed by flow cytometry 48 h after the treatment. Biological and molecular characteristics of primary B-ALL samples were identified following specific procedures in the hematological unit (Dr. Paola Ballerini, Laboratoire d’Hématologie, Assistance Publique Hôpitaux de Paris, Hôpital Armand Trousseau, Paris, France) and are described in Table S3.

### Flow cytometry

ALL and AML developments in NSG mice were characterized in BM, spleen, and PB by flow cytometry using antibodies described in Table S4. These antibodies were also used to distinguish between leukemia cells and murine cells. Apoptosis was assessed following Annexin-V-FITC (556419, BD Biosciences) staining. Binding of PKH67-Green Val-ILs was detected in the green channel. We analyzed cell subsets from PBMC samples as well as the human HSC reconstitution in PB and BM of mice, using antibodies described in Table S4. Regarding the study of mice following the injection of EL4 cells, all antibodies used to analyze the different immune cells in spleens and tumors, as well as T8 lymphocyte activation ex vivo, or the activity of T8 lymphocytes isolated ex vivo on EL4 cells, are described in Table S5. Viability was assessed using Fixable Viability Stain (FVS440UV, FVS450, and FVS510, 1:1000, BD Biosciences) or Hoechst 33342 (1:1000, Thermo Fisher Scientific). Cell subsets were analyzed using an LSR-Fortessa (BD Biosciences) or an Aurora (Cytek) apparatus. Data were analyzed using FlowJo software (V10, TreeStar Inc.). We used the Uniform Manifold Approximation and Projection (UMAP) FlowJo plugin for dimensionality reduction to visualize high-parameter datasets in a two-dimensional space. Gating strategies are shown in Fig. S11.

### Statistics

All data were expressed as means ± standard deviation (SD). Differences between the two groups were assessed with the two-tailed unpaired Student’s *t-*test or two-tailed paired Student’s *t*-test. The one-way ANOVA with Tukey’s multiple comparison tests were used to assess differences between more than two groups. Differences in Kaplan–Meier survival plots were analyzed using the Log-Rank (Mantel–Cox) test. No statistical methods were used to predetermine the sample size. No animal exclusion criteria were applied. Mice were randomly allocated to experimental groups. The variance was similar between the groups that were statistically compared. Statistics were performed using Prism 8 (GraphPad), where significance is indicated in the figures.

For more information about the proof of antibody depletion following dialysis by Western blot, the analysis of valrubicin-loaded by HPLC–MS/MS, the bisulfite conversion on DNA, PCR and pyrosequencing, the quantitative reverse transcription PCR, or the establishment of the lymphoma xenograft model through the subcutaneous injection of Raji cells and treatment with Val-ILs, see the supplementary Materials and methods online.

### Supplementary information

Supplementary data

Original data

## Data Availability

All data generated or analyzed during this study are available from the corresponding author on reasonable request.
