# Supplementary material for: Valrubicin-loaded immunoliposomes for specific vesicle-mediated cell death in the treatment of hematological cancers
Source: Cell Death Dis. 2024 May 11;15(5):328. doi: 10.1038/s41419-024-06715-5 (PMC11088660; doi:10.1038/s41419-024-06715-5)
Supplement: Supplementary file 1 — Supplementary data [file 41419_2024_6715_MOESM1_ESM.pdf]

Supplementary data

Supplementary Materials and Methods

Supplementary Figures S1 to S11

Supplementary Tables S1 to S5

Valrubicin-loaded immunoliposomes for specific vesicle-mediated cell death in the treatment of hematological cancers

Aleksandra Georgievski,[1,2] Pierre-Simon Bellaye,[1,3] Benjamin Tournier,[1,4] Hélène Choubley,[1,2,5] Jean-Paul Pais de Barros,[1,2,5] Michaële Herbst,[6] Arnaud Béduneau,[2,7] Patrick Callier,[8] Bertrand Collin,[1,3] Frédérique Végran[1,2,9] Paola Ballerini,[10] Carmen Garrido,[1,2,9,11] and Ronan Quéré.[1,2,*]

[1] Center for Translational and Molecular Medicine, UMR1231 Inserm/Université de Bourgogne, Dijon, France
[2] LipSTIC Labex, Dijon, France.
[3] Plateforme d'imagerie et de radiothérapie précliniques, Centre Georges François Leclerc-Unicancer, Dijon, France
[4] Service de Pathologie, CHU Dijon-Bourgogne, Dijon, France
[5] Plateforme DiviOmics, UMS58 Inserm BioSanD, Université de Bourgogne, Dijon, France.
[6] Laboratoire Interdisciplinaire Carnot de Bourgogne, UMR 6303 CNRS/Université de Bourgogne, Dijon, France
[7] Université de Franche-Comté, EFS, Inserm, UMR1098 RIGHT, Besançon, France
[8] Laboratoire de Génétique Chromosomique et Moléculaire, CHU Dijon-Bourgogne, Dijon, France
[9] Centre Georges François Leclerc-Unicancer, Dijon, France
[10] Laboratoire d'Hématologie, Assistance Publique-Hôpitaux de Paris, Hôpital Armand Trousseau, Paris, France
[11] Label of excellence from la Ligue Nationale contre le Cancer.

Supplementary Materials and Methods

Antibody depletion following dialysis revealed by western blot

To study the presence of IgG and αCD19 antibodies on ILs following dialysis, Laemmli buffer was applied to ILs, and for each condition the same volume of ILs was loaded on 14% sodium dodecyl sulfate–polyacrylamide gel electrophoresis (SDS-PAGE). Following gel migration, antibodies were transferred to polyvinylidene fluoride (PVDF) membranes. We used a secondary anti-mouse antibody, conjugated with horseradish peroxidase (1:5,000, Cell Signaling Technology) to detect IgG and αCD19 antibodies. Chemiluminescence was performed (Chemidoc, Bio-Rad), after applying ultra-sensitive enhanced chemiluminescent (ECL) substrate (SuperSignal West Femto Maximum Sensitivity, Thermo Fisher Scientific). Protein sizes were controlled by a protein ladder (Page Ruler Plus Prestained Protein Ladder, Thermo Fisher Scientific), and protein expression levels were assessed using ImageJ (NIH).

Analysis of valrubicin-loaded ILs-αCD19 by LC-MS/MS

ILs loaded with valrubicin (90 µL) were mixed with 10 µL of acetonitrile containing 10 ng of daunorubicin, used as an internal standard. An external calibration curve was prepared with valrubicin standards, ranging from 15 to 480 ng and mixed with 10 ng of daunorubicin, in a final volume of 100 µL. Samples and calibrants (1 µL) were injected into a 1200LC system coupled to a 6460-QqQ MS/MS system equipped with an ESI source (Agilent Technologies). Separation was achieved on a Zorbax SB-C18, 2.1×50 mm, 1.8 µm column (Agilent Technologies) maintained at 30°C at a flow rate of 0.3 mL/min with a linear gradient of (solvent A) water, containing 0.1% formic acid and (solvent B) acetonitrile/methanol (70/30 v/v), containing 0.1% formic acid as follows : 30% B for 0.1 min, up to 100% B in 4.9 min, and maintained at 100% for 5 min. Acquisitions were performed in positive Multiple Reaction Monitoring (MRM) mode (source temperature: 300°C, nebulizer gas flow rate was 4 L/min, 20 psi, sheath gas flow 12 L/min, temperature 350°C, capillary 3,000 V, VCharging 1,000 V). Transitions used for quantification/qualification of valrubicin and daunorubicin were 746.1 to 401.2 (frag 168V, CE 37V) / 746.1 to 485.0 (frag 168V, CE 25V) and 528.1 to 321.0 (frag 128V, CE 25V) / 528.1 to 363.0 (frag 128V, CE 13V), respectively. Data analysis was performed using MassHunter Workstation build 9.0.9037.0 software (Agilent Technologies).

Transmission electron microscopy (TEM)

For TEM, EVs morphology was observed by negative staining. Five microliters of ILs sample were applied to glow-discharged, carbon-coated Formwar/Carbon 200-Mesh, Cu grids (Electron Microscopy Sciences) and incubated for 1 min. Grids were then stained using UranyLess TEM Stain (Electron Microscopy Sciences) for 1 min. Visualizations were performed using a TEM (HITACHI HT7800) operating at 80kV and equipped with AMT cameras (AMT, Woburn, USA). TEM was performed by the DImaCell Imaging Facility (INRAE, Université Bourgogne, Dijon).

Establishment of the lymphoma xenograft model and treatment with ILs

Raji cells (CCL-86, ATCC) were cultured in RPMI-1640 media (Dominique Dutscher) supplemented with 10% fetal bovine serum (Dominique Dutscher) and Penicillin-Streptomycin-Amphotericin (PSA, Pan Biotech). ILs-IgG and ILs-αCD19 were tested on Raji cells *in vitro* prior to establishment of the lymphoma xenograft model in NSG mice. For the *in vivo* study, 5×10^5 Raji cells, in a volume of 100 µL of physiological saline solution, were injected subcutaneously on the body side of NSG mice. For injection, males and females were shaved and randomly assigned to experimental groups; no blinding method was used for injection. ILs were injected into the tail vein, three times every five days (days 15, 20 and 25), at a dose of 10^{11} particles in 300 µL of physiological saline solution. Tumor growth was measured over time. Mice were euthanized when the tumor reached a size limit of 500 mm^3, 30 days after the injection of Raji cells. The presence of Raji cells in the tumor was assessed by immunohistochemistry at day 30. Mice were also injected with $PKH67^+$ ILs (10^{11} particles) and sacrificed 24 hours later so that the presence of UVs in tumors could be analyzed by flow cytometry, as well as the binding of fluorescent ILs on Raji cells. Twenty-four hours after the injection of $PKH67^+$ ILs, tumors were chopped up into 3 to 4 mm pieces with a sterile scalpel. Tumor samples were then placed in 2.5 mL of the dissociation buffer, containing 60U/mL of Collagenase, Type 1 (CLS-I, LS004194, Cell Systems), 30 U/mL of Collagenase, Type 2 (CLS-II, LS004174, Cell Systems), 60 U/mL of Collagenase, Type 4 (CLS-IV, LS004186, Cell Systems), 25 µg/mL of DNAse 1 (04536282001, Merck), in filtered PBS 1×. The mixture was incubated under agitation at 37°C for 45 min. Cell suspension was filtered through 30 µm separation filters (130-041-407, Miltenyi Biotech) and centrifuged at 500 *g*. Cells were recovered to detect $PKH67^+$ ILs binding, by flow cytometry. The supernatant was centrifuged at 10,000 *g* to remove debris, then UVs were isolated from the supernatant, by pull-down, with a specific kit (15254394, Thermo Fisher Scientific) and analyzed by flow cytometry.

Bisulfite conversion, PCR and pyrosequencing

DNA was extracted from BM samples using phenol-chloroform-isoamyl alcohol (25:24:1, v/v) extraction (Thermo Fisher Scientific) and ethanol precipitation. To quantify DNA methylation level of the *CD19* gene promoter, selective conversion of nonmethylated cytosine to uracil was performed by DNA treatment with a kit for bisulfite conversion (59824, Qiagen). PCR and sequencing primer were designed using PyroMark Assay Design Software (Qiagen), on seven identified CpG sites. They were amplified and biotinylated by PCR with the following primers, forward; TAGGGAGGAGGTAAGTGTTG and reverse; TCCCTTTCTACCCTTTAAACATACC with a 5' Biotin C6. Single biotinylated DNA strands were then isolated, with Streptavidin sepharose beads (17511301, Cytiva), using the PyroMark Q24 Vacuum Workstation (Qiagen) and released into the pyrosequencing reaction plate containing sequencing primers, seq1; TTGGAGAATGGGGTT, seq2; TTTTTTTGGGGGGAT and seq3; GGTTTTTGTTTGTTTTAGTATT. After primer annealing (at 80°C for 2 min), the plate and the PyroMark Cartridge containing PyroMark Gold reagents (970902, Qiagen) were placed into the pyrosequencing instrument (PyroMark Q24, Qiagen). Analysis was performed with PyroMark Q24 2.0.7 software.

Quantitative reverse transcription PCR

On BM cells isolated from PDX mice, we performed the mRNA organic extraction with Qiazol (Qiagen). After DNAse 1 digestion (Thermo Fisher Scientific), Moloney murine leukemia virus reverse transcriptase (M-MLV RT, Promega) was used to synthesize cDNA with random primers (Promega). The following primers were then used for real-time quantitative polymerase chain reaction (qPCR): *CD19*-forward; AGAGATATGTGGGTAATG and *CD19*-reverse; ACGGTGACAATAATACTT, as well as *ACTB*-forward; CCTCGCCTTTGCCGATCC and *ACTB*-reverse; ATCATCATCCATGGTGAGCTGG. We used SYBR green qPCR Master Mix (Thermo Fisher Scientific). Experiments were carried out using the Viia7 system (Applied Biosystems). Analyses were performed according to the $2^{-\Delta\Delta Ct}$ method.

Supplementary Figures

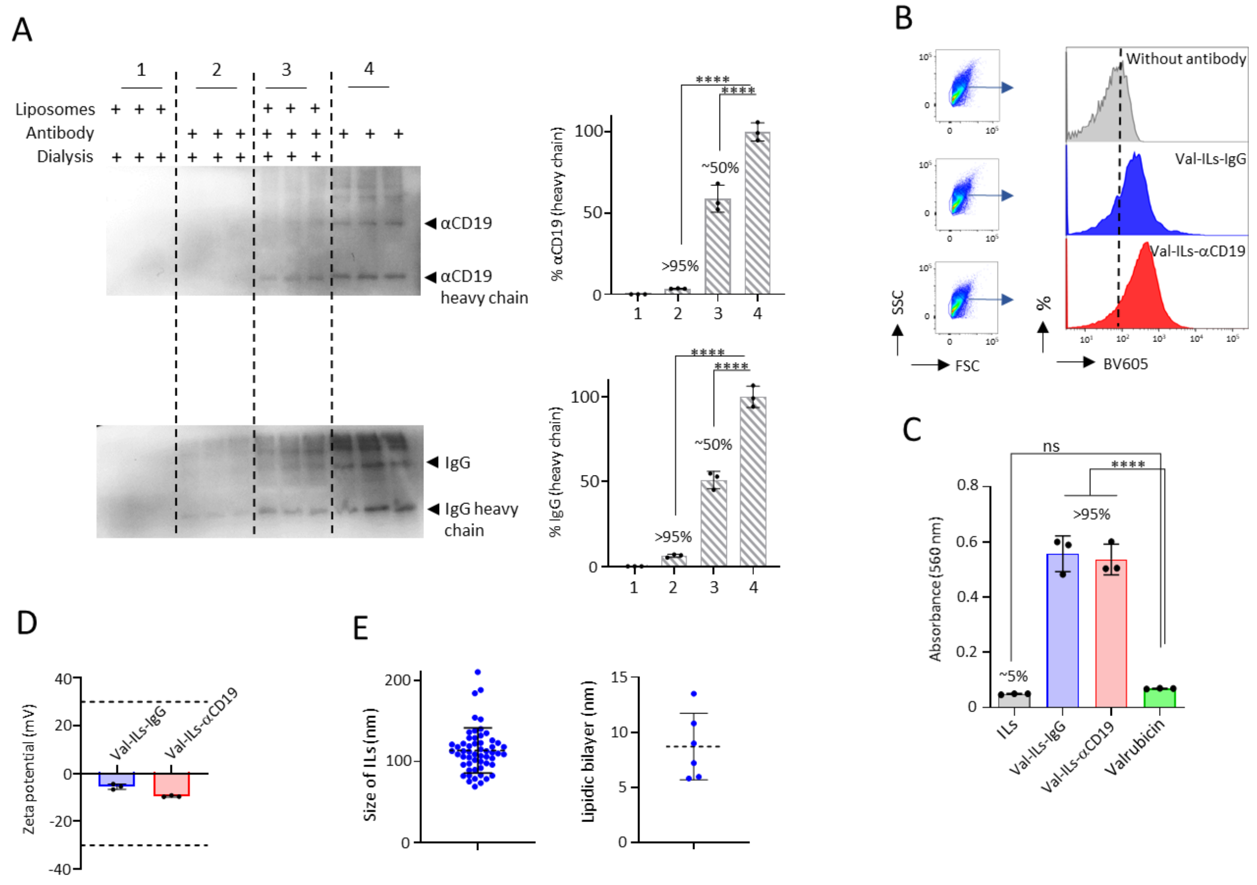

Figure S1: Development of Val-ILs-αCD19 to specifically target cells expressing CD19. (**A**) Western blot showing that following the preparation of Val-ILs and dialysis, ~50% had bound antibodies (lines 4 were compared to lines 3) and dialysis removed >95% of the unbound antibodies (lines 4 were compared to lines 2). Full length uncropped western blots for Val-ILs-IgG and Val-ILs-αCD19 (left panels) and quantification of the heavy chains (right panels). Data are normalized to lines 4 and are shown as means ± SD, *n* = 3 biological replicates. *P* value measured by one-way Anova with Tukey's multiple comparison test; $^{****}P < 0.0001$. Heavy chains at 55kDa were used for quantifications. (**B**) Flow cytometry on Val-ILs-IgG and Val-ILs-αCD19 demonstrating the presence of antibodies on their surface. Dashed line corresponding to mean fluorescence intensity obtained with Val-ILs without antibody. (**C**) Absorbance of valrubicin (560nm) showing that >95% of the non-encapsulated valrubicin was removed following dialysis of Val-ILs-IgG and Val-ILs-αCD19. Data compared to valrubicin non-encapsulated in Val-ILs after dialysis (green color). Data are shown as means ± SD, *n* = 3 biological replicates. *P* value measured by one-way Anova with Tukey's multiple comparison test; $^{****}P < 0.0001$; ns, non-significant. (**D**) Zeta potential indicating stability of Val-ILs-IgG and Val-ILs-αCD19. Data are shown as means ± SD, *n* = 3 biological replicates. Dashed bars corresponding to recommended stability limits (between -30 mV and +30 mV). (**E**) Val-ILs-αCD19 observed by TEM have an average size of 114 ± 28 nm and the lipid bilayer has an average size of 7.7 ± 3.0 nm.

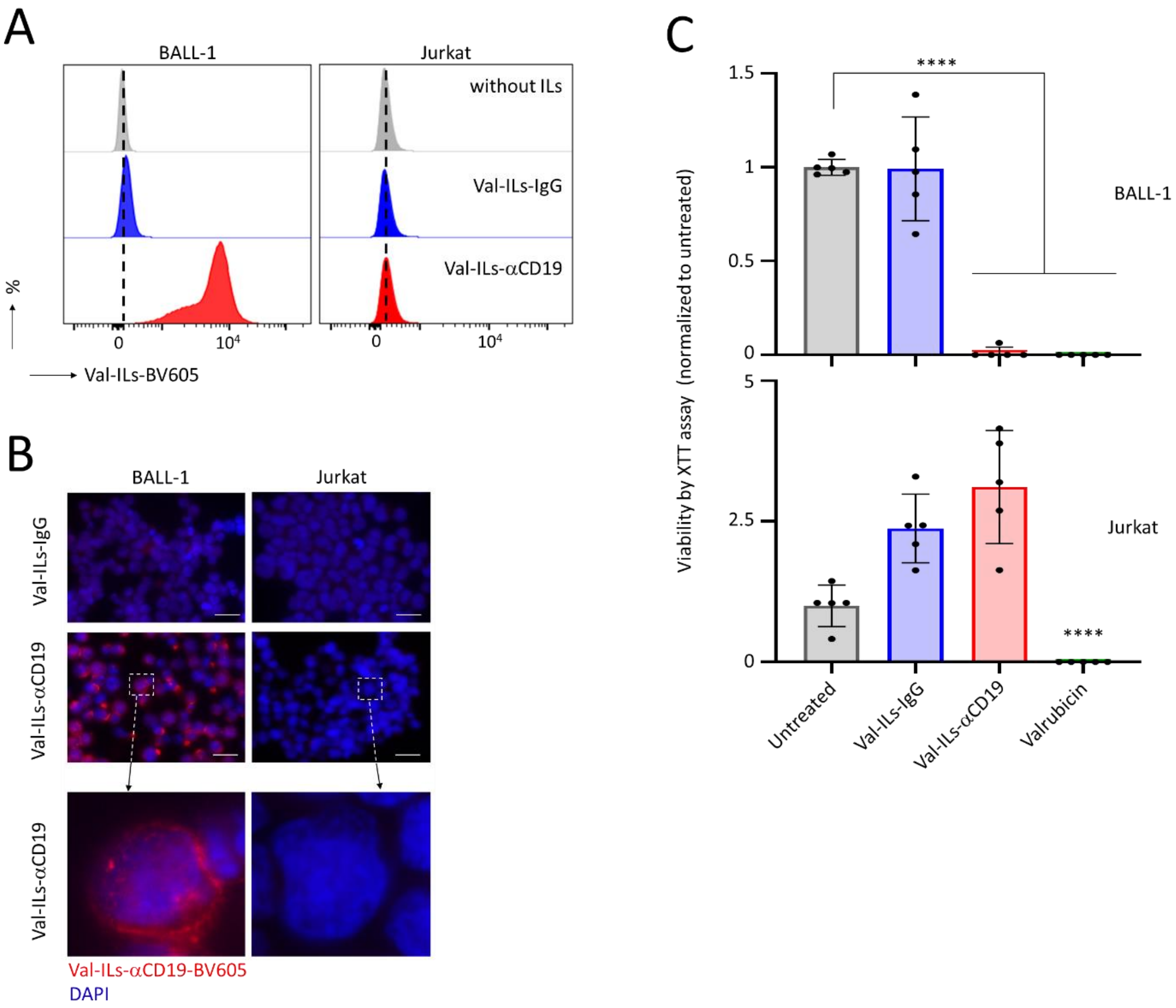

Figure S2: Val-ILs efficiently affect the viability of CD19-expressing cells. (**A**) Flow cytometry demonstrating that BALL-1 cells bound Val-ILs-αCD19 but not the Jurkat cells. (**B**) Microscopy by fluorescent immunostaining on BALL-1 and Jurkat cells treated with 2,000 Val-ILs-IgG or Val-ILs-αCD19/cell, after one hour, magnification ×63, scale bar represents 10 µm. Val-ILs-αCD19 specifically targeted BALL-1 cells, but not the Jurkat cells, and Val-ILs-IgG did not bind to both cell lines. (**C**) Viability assessed on BALL-1 and Jurkat cells, treated with Val-ILs-IgG or Val-ILs-αCD19 (2,000 particles/cell) or valrubicin (1 µM). XTT assay performed after 72 hours of treatment. Val-ILs-αCD19 (2,000 particles/cell) efficiently affected the viability of BALL-1 cells to a level that is similar to 1 µM valrubicin, and Val-ILs-IgG did not affect the viability of BALL-1 cells, and neither Val-ILs-IgG nor Val-ILs-αCD19 affected the Jurkat cells' viability. Data normalized to untreated controls; $n = 5$ biological replicates. Data are shown as means ± SD. P value measured by one-way Anova with Tukey's multiple comparison test; ****$P < 0.0001$.

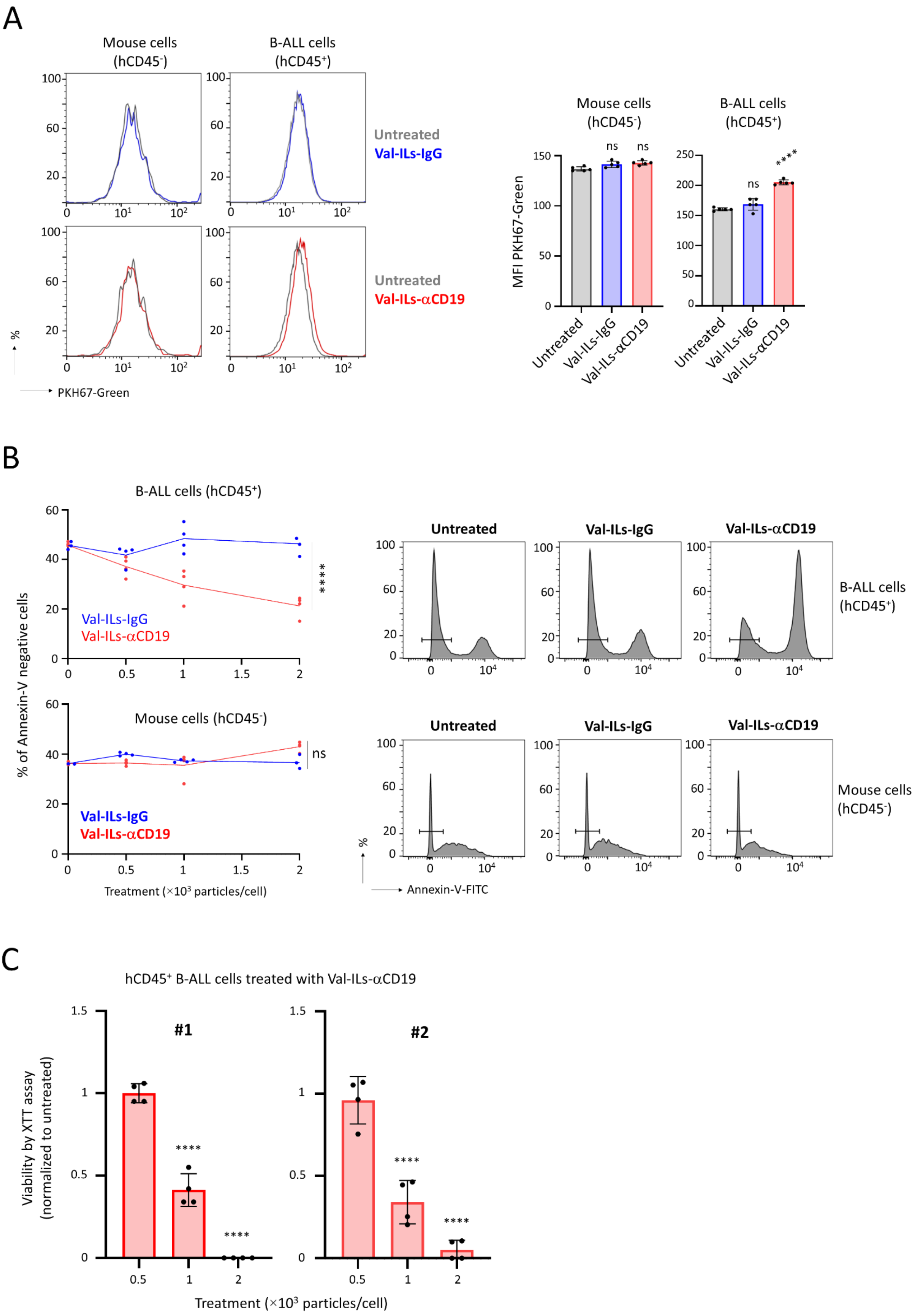

A
Mouse cells (hCD45⁻)
B-ALL cells (hCD45⁺)
Untreated
Val-ILs-IgG
Untreated
Val-ILs-αCD19
%
PKH67-Green
MFI PKH67-Green
ns
ns
ns
****
Untreated
Val-ILs-IgG
Val-ILs-αCD19
B
B-ALL cells (hCD45⁺)
% of Annexin-V negative cells
Val-ILs-IgG
Val-ILs-αCD19
****
Mouse cells (hCD45⁻)
ns
Treatment (×10³ particles/cell)
Untreated
Val-ILs-IgG
Val-ILs-αCD19
B-ALL cells (hCD45⁺)
Mouse cells (hCD45⁻)
Annexin-V-FITC
C
hCD45⁺ B-ALL cells treated with Val-ILs-αCD19
#1
#2
Viability by XTT assay (normalized to untreated)
Treatment (×10³ particles/cell)

Figure S3: Val-ILs-αCD19 affects the viability of B-ALL CD19$^+$ cells isolated from PDX mice *ex vivo*. (**A**) Flow cytometry on B-ALL cells (hCD45$^+$ hCD19$^+$) and endogenous murine BM cells (hCD45$^-$ hCD19$^-$) after 18 hours of exposure with 2,000 particles/cell, with PKH67-Green fluorescent Val-ILs-IgG or Val-ILs-αCD19. Cells were recovered from B-ALL PDX model #1, 30 days after transplantation. Mean fluorescence intensity (MFI) showing that Val-ILs-αCD19, but not Val-ILs-IgG, can target B-ALL cells (hCD45$^+$ hCD19$^+$), leaving murine BM cells untargeted. Examples of plots and statistics, n = 5 biological replicates. (**B**) Apoptosis assessed by Annexin-V staining and flow cytometry, on hCD45$^+$ B-ALL cells and hCD45$^-$ murine cells, after 48 hours of exposure with 500, 1,000 or 2,000 particles/cell. Examples of plots and statistics, n = 4 biological replicates. Val-ILs-αCD19, but not Val-ILs-IgG, induced apoptosis of B-ALL cells, leaving murine BM cells away. (**C**) Viability was assessed on hCD45$^+$ cells isolated with magnetic beads from the BM of B-ALL PDX mice (models #1 and #2) by XTT assay, following a single treatment at day 0, with 500, 1,000 or 2,000 particles/cell. XTT was assessed after 72 hours of treatment and data were normalized to untreated controls, n = 4 biological replicates. On this figure, data are shown as means ± SD. (A, C) P value measured by one-way Anova with Tukey's multiple comparison test. ****$P < 0.0001$; ns, non-significant. (B) P value measured by two-tailed unpaired Student's t test; ****$P < 0.0001$; ns, non-significant.

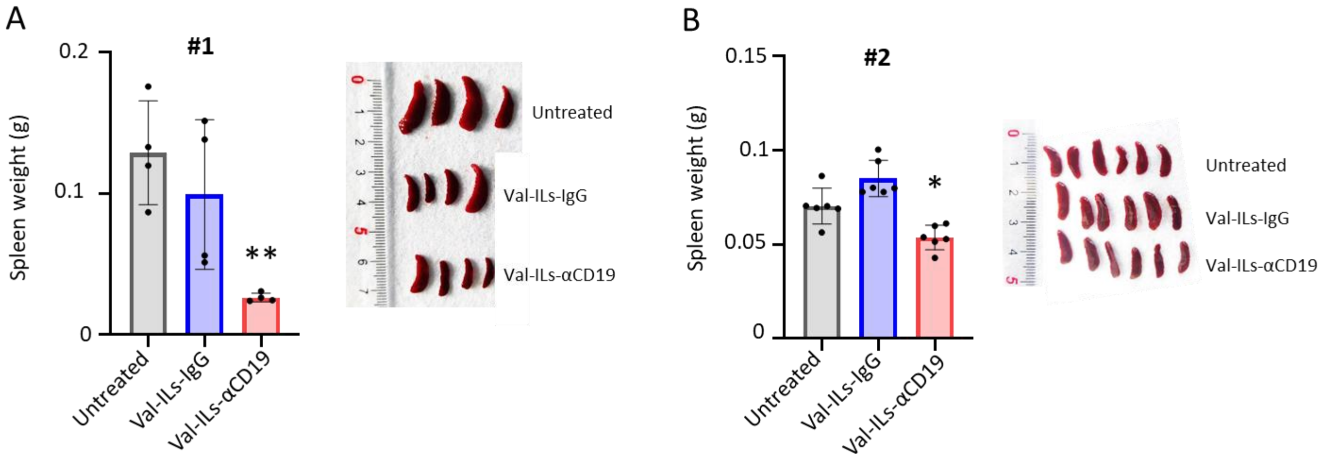

Figure S4: Reduced splenomegaly is observed in mice treated with Val-ILs-αCD19. PDX mice were treated with three injections of 10^{11} Val-ILs, on days 15, 20 and 25 post-transplantation. Weight and pictures of spleens on day 35, *n* = 4 mice per group for PDX #1 (**A**) and *n* = 6 mice per group for PDX #2 (**B**). Data are shown as means ± SD. *P* value calculated against untreated condition and measured by one-way Anova with Tukey's multiple comparison test; $^{*}P < 0.05$; $^{**}P < 0.01$.

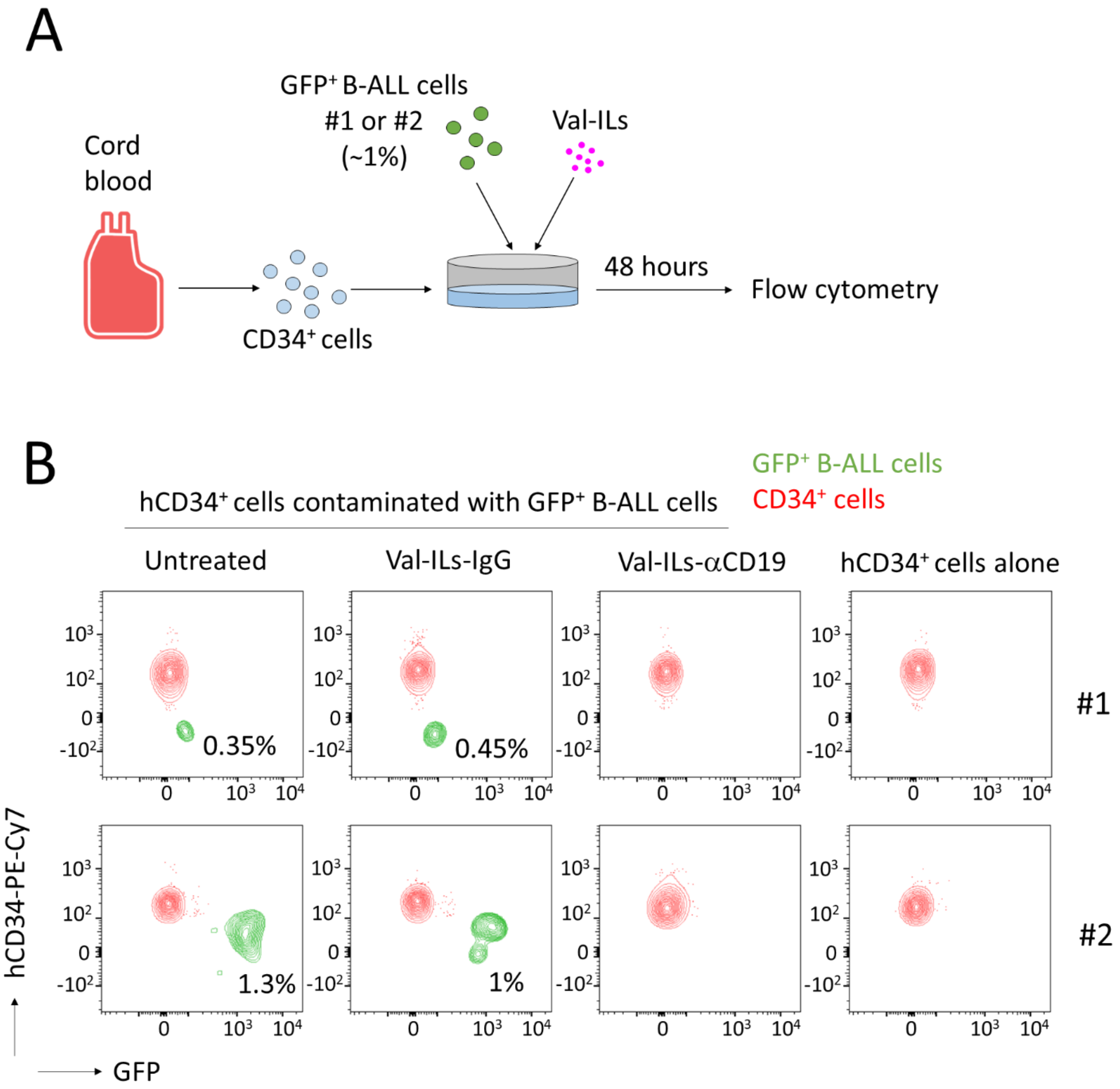

Figure S5: Val-ILs-αCD19 eradicate malignant B-ALL cells among CD34+ HSC. (**A**) Description of the procedure to deliberately contaminate CD34+ HSC isolated from human cord blood with ~1% of GFP+ B-ALL cells (isolated from PDX #1 or #2). (**B**) After 48 hours of *in vitro* treatment, with Val-ILs-IgG or Val-ILs-αCD19 (2,000 particles/cell), cells were analyzed by flow cytometry. Val-ILs-αCD19 eliminated efficiently 100% of the B-ALL cells, while viability of the CD34+ cells remained unaffected. Data were gated on viable FVS440UV negative cells.

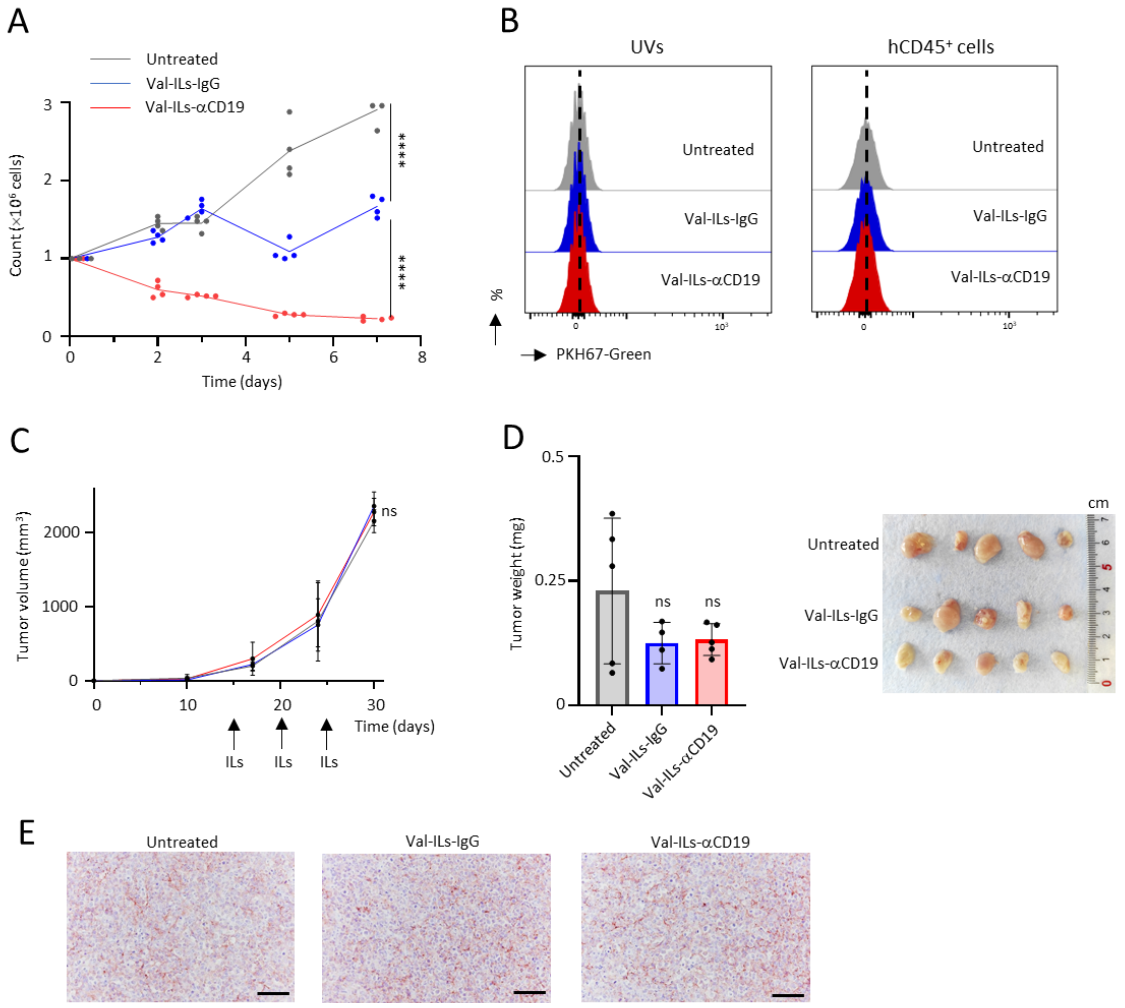

Figure S6: Val-ILs-αCD19 have no effect on lymphoma xenografted in mice. (**A**) Trypan blue was used to assess the number of viable Raji cells over time following a single treatment of 2,000 particles/cell with Val-ILs-IgG or Val-ILs-αCD19, *n* = 4 biological replicates. (**B**) NSG mice were subcutaneously injected with 5×10^5 Raji cells, on the body side. Val-ILs labeled with PKH67-Green were i.v. injected at 10^{11} particles in NSG mice 20 days after transplantation of Raji cells, then, 18 hours later, UVs were purified from the tumors to assess detection of PKH67$^+$ Val-ILs and binding of green fluorescent Val-ILs on hCD45$^+$ Raji cells in the tumors by flow cytometry. Cytometry plots showing no detection of PKH67$^+$ Val-ILs among UVs, or Raji cells positive for PKH67$^+$ Val-ILs binding. (**C**) Val-ILs were injected into the tail vein three times every five days (days 15, 20 and 25; arrows), at a dose of 10^{11} particles, in 300 µL of physiological saline solution. Tumor growth volume was assessed over time in PDX mice, *n* = 5 mice per group. (**D**) Tumor weight at day 30, when mice were euthanized, showing no significant variation between groups, *n* = 5 mice per group. (**E**) Immunohistochemistry, following staining with hCD19 antibody on tumor sections, showing detection of many Raji cells (brown cells), for all conditions. Counterstaining of cells in tumors was obtained by Giemsa staining. Data are representative of four mice, magnification ×20, black scale bars represent 100 µm. On this figure, data are shown as means ± SD. *P* value measured by one-way Anova with Tukey's multiple comparison test; $^{****}P < 0.0001$; ns, non-significant.

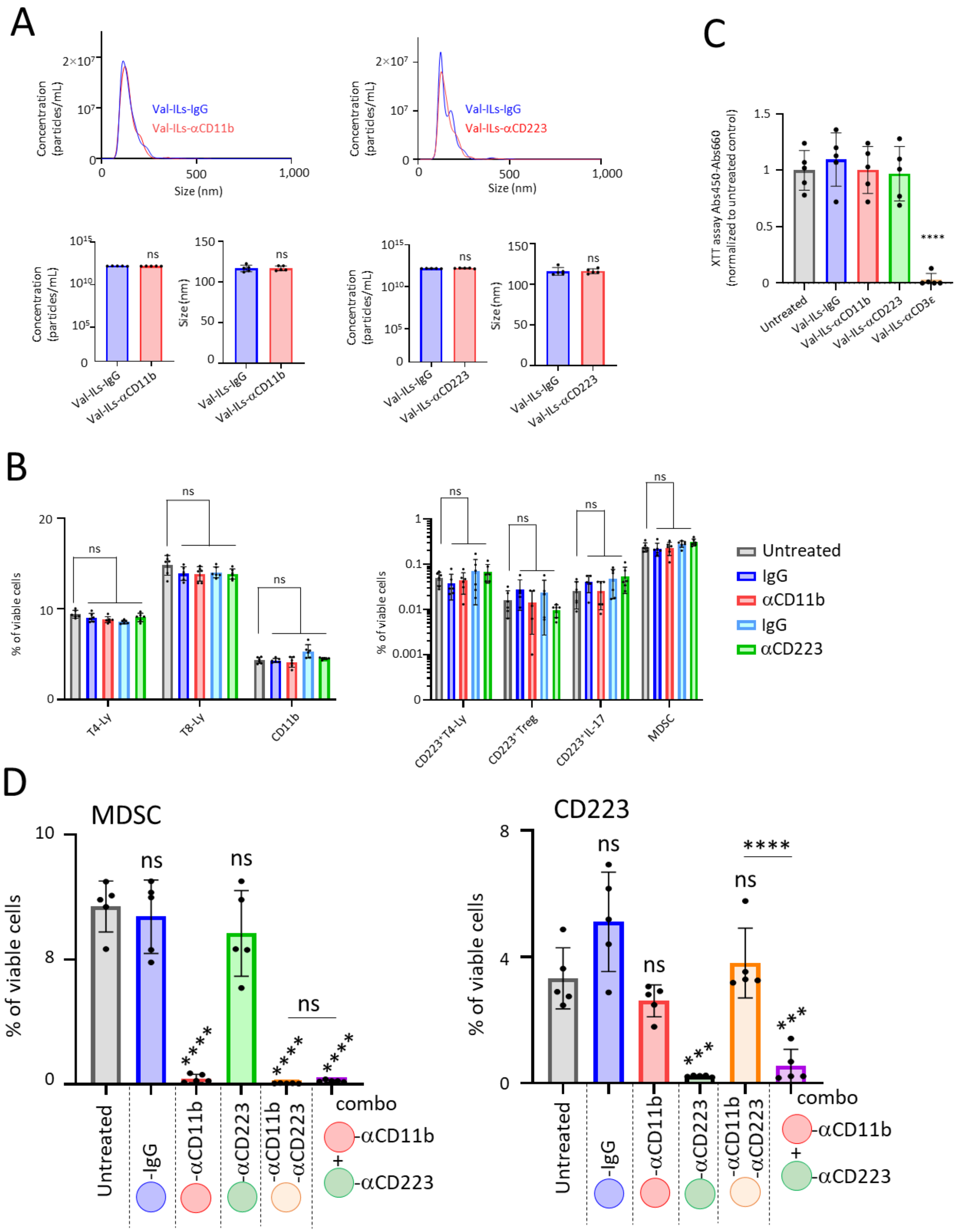
A
Concentration (particles/mL)
Val-ILs-IgG
Val-ILs-αCD11b
Size (nm)
Val-ILs-IgG
Val-ILs-αCD223
Size (nm)
Concentration (particles/mL)
ns
Size (nm)
ns
Val-ILs-IgG
Val-ILs-αCD11b
Val-ILs-IgG
Val-ILs-αCD11b
Concentration (particles/mL)
ns
Size (nm)
ns
Val-ILs-IgG
Val-ILs-αCD223
Val-ILs-IgG
Val-ILs-αCD223
C
XTT assay Abs450-Abs660 (normalized to untreated control)
****
Untreated
Val-ILs-IgG
Val-ILs-αCD11b
Val-ILs-αCD223
Val-ILs-αCD3ε
B
% of viable cells
ns
ns
ns
T4-Ly
T8-Ly
CD11b
% of viable cells
ns
ns
ns
ns
CD223+T4-Ly
CD223+Treg
CD223+IL-17
MDSC
Untreated
IgG
αCD11b
IgG
αCD223
D
MDSC
% of viable cells
ns
ns
****
ns
****
****
Untreated
-IgG
-αCD11b
-αCD223
-αCD11b
-αCD223
combo
-αCD11b
+
-αCD223
CD223
% of viable cells
ns
****
ns
ns
***
***
Untreated
-IgG
-αCD11b
-αCD223
-αCD11b
-αCD223
combo
-αCD11b
+
-αCD223

Figure S7: Development of Val-ILs targeting immune repressive cells for lymphoma immunotherapy. (**A**) Quantification and size of Val-ILs measured by NTA for Val-ILs-αCD11b and Val-ILs-αCD223, *n* = 5 biological replicates for each condition. Data are shown as means ± SD. *P* value measured by two-tailed unpaired Student's *t* test; ns, non-significant. (**B**) Antibodies used to prepare Val-ILs have no cytotoxic effect on murine immune cells. Six days following the transplantation of EL4 cells, spleens were recovered and following hemolysis the immune cells were treated with an αCD11b, αCD223 or the IgG control antibodies. Viability of cells was assessed by flow cytometry, 48 hours after treatment with the antibodies used to prepare Val-ILs. This experiment shows that all antibodies used in this study, when non-incorporated on the surface of Val-ILs have no direct cytotoxic effect on immune cells, *n* = 6 mice. Data are shown as means ± SD. *P* value calculated against untreated and measured by one-way Anova with Tukey's multiple comparison test; ns, non-significant. (**C**) Val-ILs have no direct cytotoxic effect on EL4 cells. Viability of EL4 cells was assessed by XTT assay, 48 hours after treatment with Val-ILs, *n* = 5 biological replicates. Val-ILs-αCD3ε were used as positive control, while EL4 cells expressed CD3ε. Data are shown as means ± SD. *P* value calculated against untreated and measured by one-way Anova with Tukey's multiple comparison test; ****$P < 0.0001$; no statistic is shown when *P* value is non-significant. (**D**) Six days following the transplantation of EL4 cells, spleens were recovered and following hemolysis the immune cells were treated with Val-ILs-IgG, Val-ILs-αCD11b, Val-ILs-αCD223, Val-ILs loaded with αCD11b and αCD223, or a combination (combo) of Val-ILs-αCD11b and Val-ILs-αCD223. For all conditions, 2,000 particles/cell were administrated. Viability was assessed by flow cytometry on CD11b and CD223 cells, following 3 days of treatment, *n* = 5 biological replicates. Data are shown as means ± SD. *P* value calculated against untreated and measured by one-way Anova with Tukey's multiple comparison test; ***$P < 0.001$; ****$P < 0.0001$; ns, non-significant.

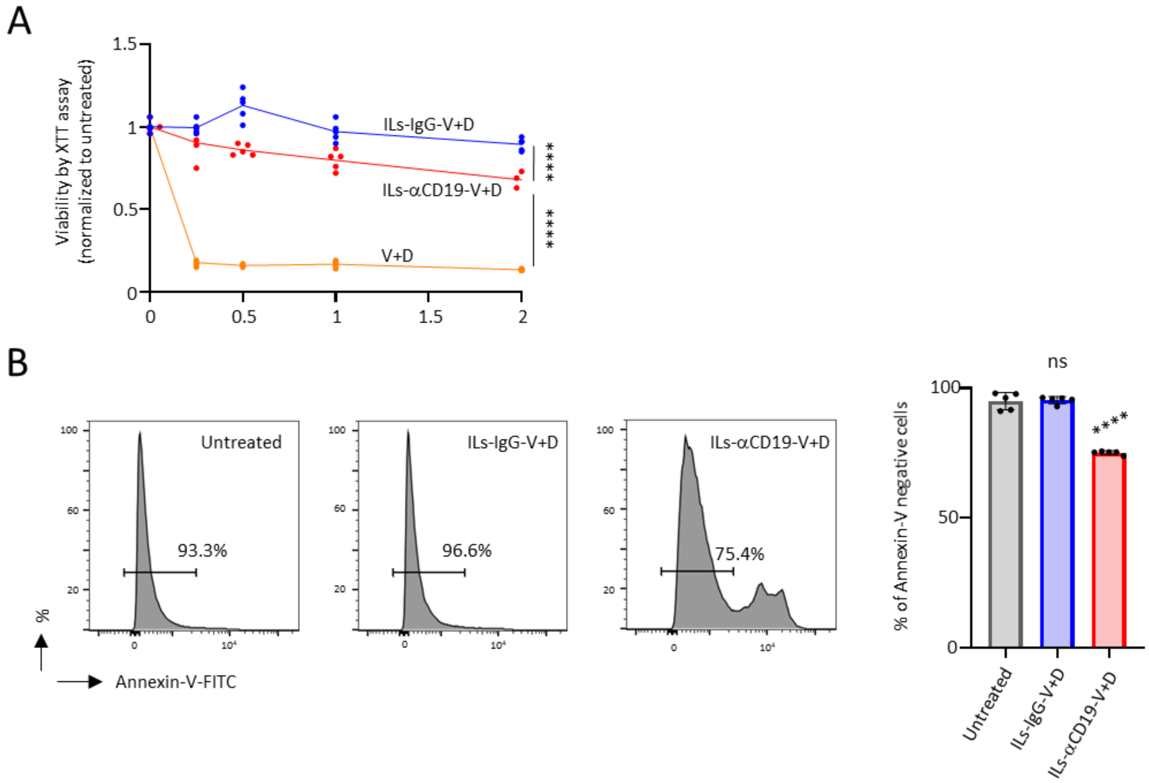

Figure S8: ILs-αCD19 loaded with Vincristine and Dexamethasone have lower efficiencies. We prepared ILs encapsulating Vincristine and Dexamethasone in the internal aqueous compartment of the vesicles during the sonication process. (**A**) Viability following XTT assay, 72 hours after treatment with an increasing amount of ILs (250, 500, 1,000 and 2,000 particles/cell) or a combination of 1 µM Vincristine and 1 µM Dexamethasone (V+D). Data are normalized to untreated controls, *n* = 5 biological replicates. *P* value measured by one-way Anova with Tukey's multiple comparison test for 2,000 particles/cell; *****P* < 0.0001. ILs incorporated with these drugs showed a lower efficiency in their capacity to induce death of BALL-1 cells, and treatment with 2,000 particles/cell induced a drop in viability for only ~25% of BALL-1 cells. (**B**) Annexin-V immunostaining and flow cytometry, 72 hours after treatment with 2,000 particles/cell. Examples of cytometry datasets are shown on the left panel. Statistics are shown on the right panel. Data are shown as means ± SD, *n* = 5 biological replicates. *P* value measured by one-way Anova with Tukey's multiple comparison test; *****P* < 0.0001; ns, non-significant.

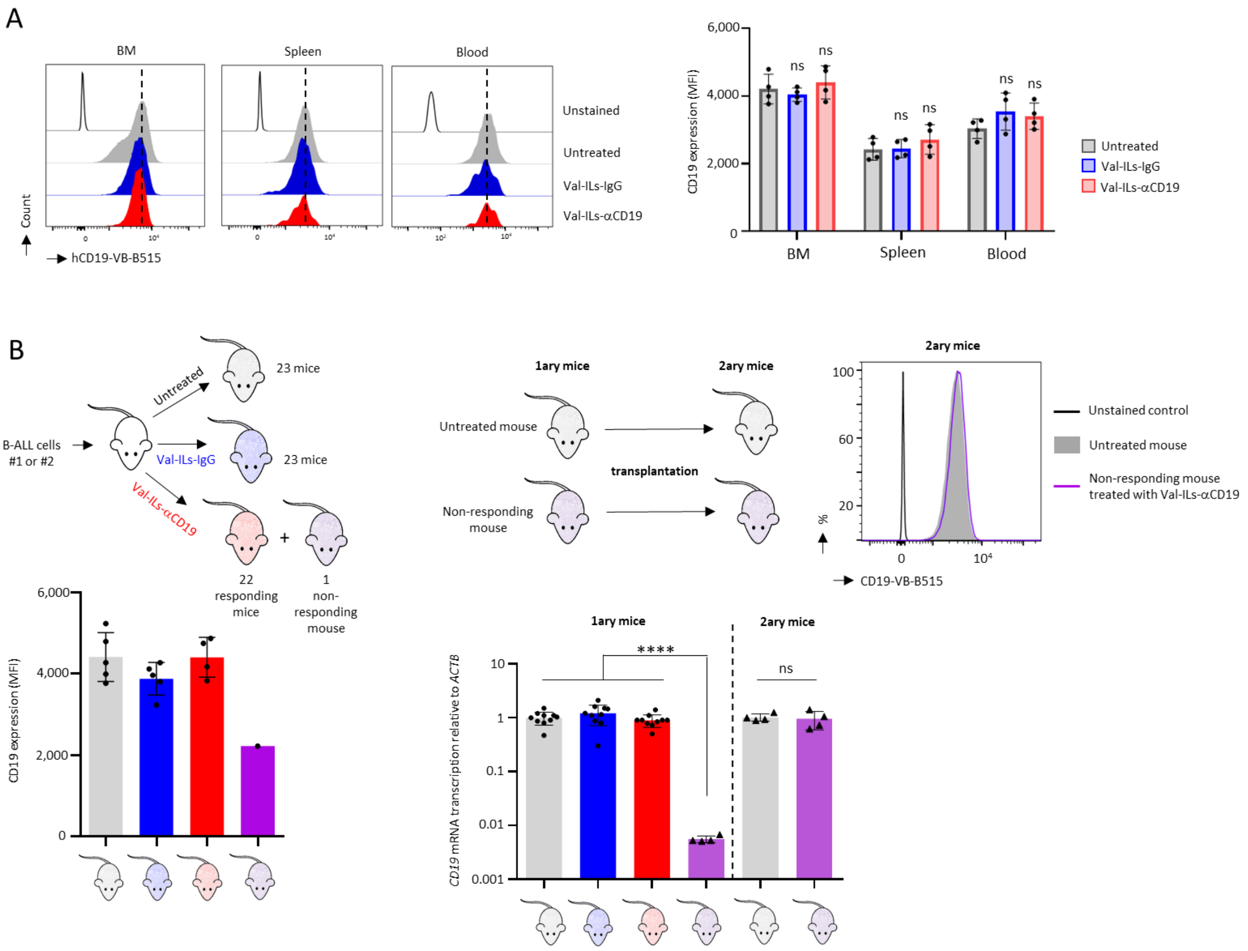

C

	Sequence	Primer	
PCR	TAGGGAGGAGGTAAGTGTTGTGAGTTTGGAGGGTTTTTGGAGAATGGGGTTTGAGGYGTGATTATY GTTTTTTTTTTGGGGGGATTGTTTGTYGTTTTYGTAGATATTTATGGTTGAGTGTTTTTTAGGTTTTTG TTTGTTTTAGTATTTTTTGYGYGAAGTTGGGTGTTTYGGAGAGTTTGATTATTATGTTATTTTTTYGTTT TTTTTTTTTTTTTTTTTTTTTTTATTTTTATGGAAGTTAGGTTYGAGGAATTTTTAGTGGTGAAGGTGGAA GGTATGTTTAAAGGGTAGAAAGGGA	Forward<br>Reverse	TAGGGAGGAGGTAAGTGTTG<br>TCCCTTTCTACCCTTTAAACATACC
CpG 1-2	TGAGGYGTGATTATYGTTTTTTTTTTGGGGGGATTGTTT	Seq1	TTGGAGAATGGGGTT
CpG 3-4	TGTTTGTYGTTTTYGTAGATATTTATGGTTGAGTGTTTT	Seq2	TTTTTTTGGGGGGAT
CpG 5-7	TTTTGYGYGAAGTTGGGTGTTTYGGAGAGTTTGATTATTATGTTATTT	Seq3	GGTTTTTGTTTGTTTTAGTATT

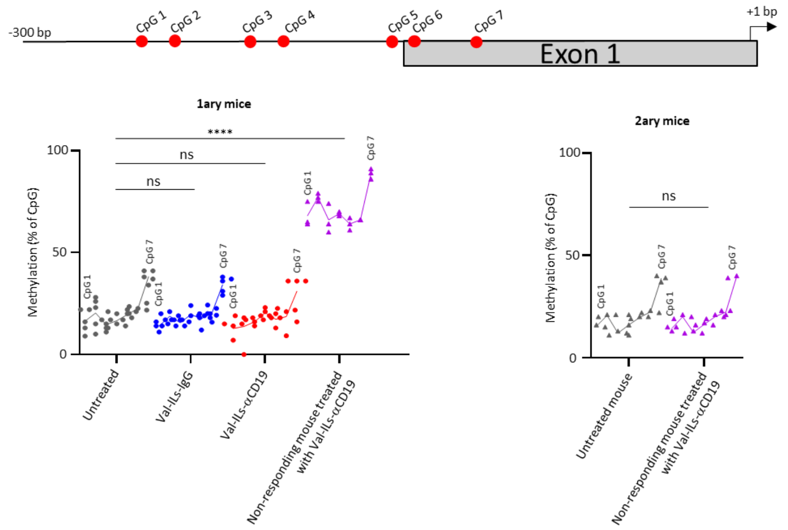

Figure S9: Hypermethylation of the CD19 promoter was the cause of antigen-negative escape from one mouse treated with Val-ILs-αCD19. (**A**) Residual cells analyzed from mice treated with Val-ILs-αCD19 still expressed CD19. Mice were treated with three injections of 10^{11} Val-ILs, on days 15, 20 and 25 post-transplantation. (**A**) Example of cytometry dataset obtained by flow cytometry on B-ALL cells, in BM, spleen and blood of mice at day 35 post transplantation. Dashed line corresponds to mean fluorescence intensity (MFI) obtained with untreated control. Statistics of the CD19 expression on B-ALL cells, in BM, spleen and blood, for PDX model #1, at day 35 post transplantation, n = 4 biological replicates. (**B**) Few antigen-negative escapes to Val-ILs-αCD19 observed *in vivo*. Left panel; following the *in vivo* treatment of 23 mice (#1 and #2) with Val-ILs-αCD19, only one single mouse did not respond and showed an elevated level of B-ALL cells in the BM. Flow cytometry showing that the leukemia cells in this animal had lower CD19 protein expression, while the residual B-ALL cells in all the responding mice displayed a level of CD19 expression comparable to untreated mice or mice treated with the Val-ILs-IgG, n = 5 untreated mice, n = 5 mice treated with Val-ILs-IgG, n = 4 responding mice and n = 1 non-responding mouse. Upper right panel; following the transplantations of cells from primary mice (1ary) to secondary (2ary) mice, the lower expression of the CD19 antigen was not maintained at the protein level, as assessed by flow cytometry. Bottom right panel; in this non-responding mouse (1ary mouse), the *CD19* gene showed a lower transcription rate, measured by RTqPCR, and this *CD19* transcription reached a normal level following transplantation (2ary mouse), n = 10 mice, n = 4 technical replicates (Δ) with the non-responding mouse, in 1ary and 2ary mice. (**C**) Seven CpG sites were detected on the promoter of the *CD19* gene. Primers were designed for PCR and sequencing following conversion of nonmethylated cytosine to uracil performed by DNA treatment with sodium bisulfite. The non-responding primary mouse showed hypermethylation of the seven CpGs, n = 3 technical replicates (Δ), compared with mice from the other groups; n = 5 mice for untreated, n = 5 mice for Val-ILs-αIgG, n = 4 mice for Val-ILs-αCD19. Following the transplantations of cells from primary mice (1ary) to secondary (2ary) mice, this hypermethylation of the *CD19* promoter was not maintained, n = 3 technical replicates (Δ) for the untreated mouse, as well as the non-responding mouse. In conclusion, further CpG were detected on the CD19 promoter. We discovered that, only in this non-responding mouse, hypermethylation of the CD19 promoter enabled the antigen-negative escape to Val-ILs-αCD19. This CD19 hypermethylation reached a normal level following transplantation in a secondary mouse. On this figure, data are shown as means ± SD. P value measured by one-way Anova with Tukey's multiple comparison test; ****$P < 0.0001$; ns, non-significant.

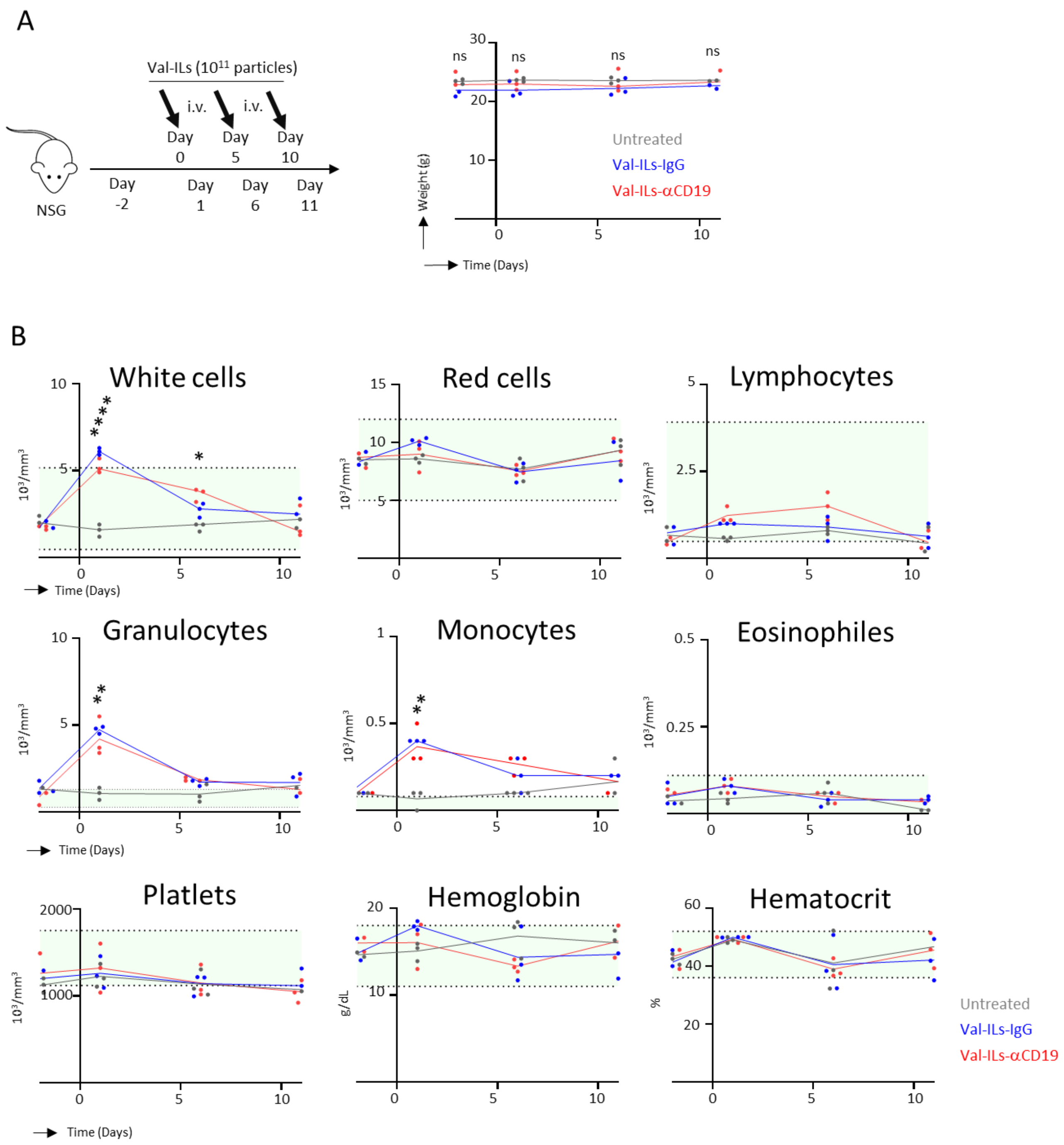

Figure S10: Injection of Val-ILs does not disturb mice' weight overtime and presents no toxicity on blood parameters. (**A**) Procedure followed to treat mice with Val-ILs and measure mice weight overtime. Weights measured following the i.v. injection of Val-ILs, data showing means, *n* = 3 mice per group. *P* value measured by one-way Anova with Tukey's multiple comparison test; ns, non-significant. (**B**) Data showing that the i.v. injections of Val-ILs have few effects on blood parameters. Increase levels of white cells, granulocytes and monocytes were observed after the first injection, following the i.v. injections of Val-ILs-αCD19 or Val-ILs-IgG, data showing means, *n* = 3 mice per group. *P* value calculated against the untreated condition and measured by one-way Anova with Tukey's multiple comparison test; *$P < 0.05$; **$P < 0.01$; ****$P < 0.0001$. No statistic was shown when non-significant

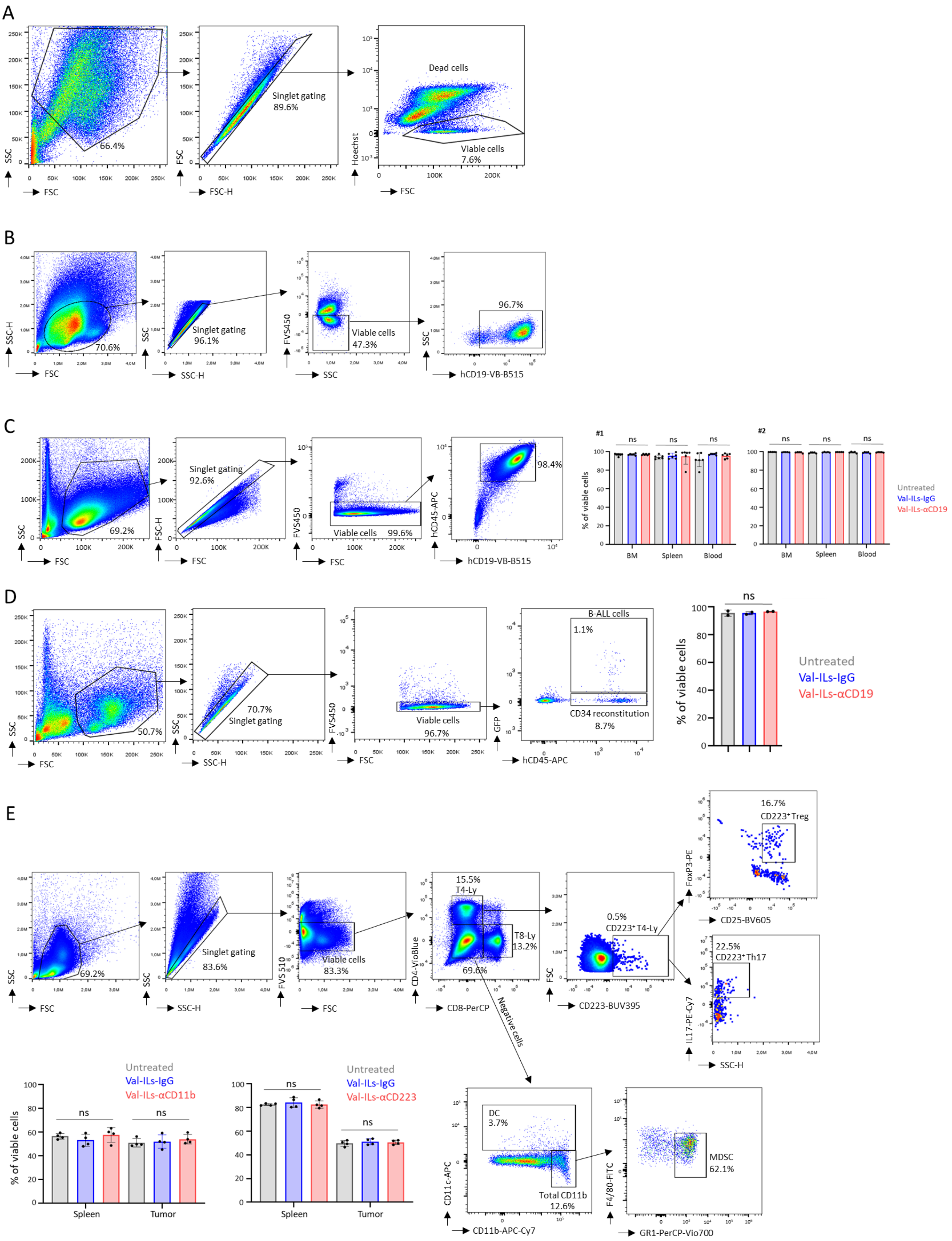

A
66.4%
Singlet gating
89.6%
Dead cells
Viable cells
7.6%
SSC
FSC
FSC-H
Hoechst
B
70.6%
Singlet gating
96.1%
Viable cells
47.3%
96.7%
SSC-H
SSC
FVS450
hCD19-VB-B515
C
69.2%
Singlet gating
92.6%
Viable cells 99.6%
98.4%
FSC-H
hCD45-APC
#1
#2
ns
% of viable cells
BM
Spleen
Blood
Untreated
Val-ILs-IgG
Val-ILs-αCD19
D
50.7%
70.7%
Singlet gating
Viable cells
96.7%
B-ALL cells
1.1%
CD34 reconstitution
8.7%
GFP
hCD45-APC
E
69.2%
Singlet gating
83.6%
Viable cells
83.3%
15.5%
T4-Ly
T8-Ly
13.2%
69.6%
0.5%
CD223⁺ T4-Ly
16.7%
CD223⁺ Treg
22.5%
CD223⁺ Th17
FVS 510
CD4-VioBlue
CD8-PerCP
CD223-BUV395
FoxP3-PE
CD25-BV605
IL17-PE-Cy7
Negative cells
DC
3.7%
Total CD11b
12.6%
MDSC
62.1%
CD11c-APC
CD11b-APC-Cy7
F4/80-FITC
GR1-PerCP-Vio700
Val-ILs-αCD11b
Val-ILs-αCD223
Spleen
Tumor

Figure S11: Flow cytometry gating strategies. (**A**) Gating strategy corresponding to Fig. 1E was applied to BALL-1 cells. A total of 10^5 events were recorded across all samples shown on Fig. 1E. Statistics on viable BALL-1 cells are shown on Fig. 1E. (**B**) Gating strategy corresponding to Fig. 1G was applied to primary B-ALL samples isolated from human BM. An example from a primary sample is shown. A total of 10^6 events were recorded across all primary B-ALL samples shown on Fig. 1G. Statistics on viable primary B-ALL cells are shown on Fig. 1G. (**C**) Gating strategy corresponding to Fig. 2C was applied to samples isolated from different organs of B-ALL PDX mouse models. An example from a BM sample is shown. Statistics are shown for BM, spleen and blood, on the right panel. Data indicating that analysis of Fig. 2C was conducted using an equal number of viable cells for all samples within each group. A total of 10^6 events were recorded across all samples. (**D**) Gating strategy corresponding to Fig. 3B was applied to BM samples isolated from mice. A total of 10^6 events were recorded across all samples. An example from a BM sample is shown, with statistics on the right panel indicating that studies on Fig. 3B were conducted using an equal number of viable cells for all samples within each group. (**E**) Gating strategy corresponding to Fig. 6B and Fig. 7B, C, D was applied to cells isolated from spleens and tumors. A total of 10^6 events were recorded across all samples shown on Fig. 6B and Fig. 7B, C, D. An example from a spleen sample is shown, with statistics on the bottom left panel indicating that studies were conducted using an equal number of viable cells for all samples within each group, for Val-ILs-αCD11b on Fig. 6B and for Val-ILs-αCD223 on Fig. 7B, C, D. On this figure, data are shown as means ± SD. *P* value measured by one-way Anova with Tukey's multiple comparison test; ns, non-significant.

Supplementary Tables

Antigen	Fluorochrome	Supplier	Reference
CD19	BV605	BD Biosciences	740394
IgG1, k	BV605	BD Biosciences	562652
CD7	VioBright-FITC	Miltenyi biotech	130-105-844
IgG2b	VioBright-FITC	Miltenyi biotech	130-104-575
CD33	PE-Cy5	BD Biosciences	551377
IgG1, k	PE-Cy5	BD Biosciences	555750

Table S1: Antibody used to prepare Val-ILs for the treatment of leukemia in PDX models. List of antibodies used to prepare Val-ILs, including IgG isotypes control antibodies.

Antigen	Fluorochrome	Supplier	Reference
CD11b	APC-R700	BD Biosciences	564985
IgG2b, k	APC-R700	BD Biosciences	564984
CD233	BV650	BD Biosciences	740560
IgG1, k	BV650	BD Biosciences	563848
CD25	PE-Cy7	BD Biosciences	552880
F4/80	BV605	BD Biosciences	743281
Ly6c	VioGreen	Miltenyi biotech	130-102-207
IL-17	PE-Cy7	Biolegend	506921
PD1	VioBlue	Miltenyi biotech	130-121-437
PD-L1	BV605	Biolegend	124321
CTLA4	APC	Miltenyi biotech	130-102-518
CD3ε	PE-Cy7	BD Biosciences	552774

Table S2: Antibody used to prepare Val-ILs for the treatment of EL4 lymphoma mice. List of antibodies used to prepare Val-ILs, including IgG isotypes control antibodies.

	Sex	Age	Source	Blast (%)	Molecular characteristics	Viability		
						Untreated	ILs-IgG	ILs-αCD19
**B1**	F	14	BM	90%	TEL/AML1 (t)	100%	175%	2%
**B2**	F	6	BM	89%	TEL/AML1 (t)	100%	103%	0%
**B3**	M	5	BM	90%	TEL/AML1 (t)	100%	116%	0%
**B4**	F	13	BM	80%	BCR/ABL (t), Hyperploidy	100%	101%	2%
**B5**	F	5	BM	93%	E2A/PBX1 (t)	100%	175%	0%
**B6**	M	4	BM	90%	Hyperploidy	100%	159%	11%
**B7**	M	4	BM	85%	P2RY8/CRLF2 (d)	100%	125%	20%
**B8**	M	4	BM	87%	Hyperploidy	100%	156%	12%
**B9**	F	4	BM	75%	nd	100%	104%	8%
**B10**	F	13	BM	87%	EBF1-JAK2 (t), PAX5 (m)	100%	147%	12%
**B11**	F	6	BM	63%	IKZD1D (d), KRAS (m)	100%	166%	5%
**B12**	M	5	BM	73%	Hyperploidy	100%	100%	1%
**B13**	M	5	BM	95%	IL7R (m), NRAS (m)	100%	195%	3%

Table S3: Biological and molecular characteristics of primary B-ALL samples. B-ALL samples were isolated from the BM of thirty individuals with B-ALL (B1 to B13). Viability obtained with untreated Val-ILs-IgG or Val-ILs-αCD19, after 48 hours of treatment *ex vivo* was shown for each B-ALL sample. Translocation (t), deletion (d), mutation (m), not determined (nd), bone marrow (BM), male (M), female (F).

Antigen	Fluorochrome	Supplier	Reference	Dilution
CD11b	VioBlue	Miltenyi biotech	130-097-336	1:100
CD19	VB-B515	Miltenyi biotech	130-113-650	1:100
CD19	PE-Cy7	BD Biosciences	557835	1:100
CD3	VioGreen	Miltenyi biotech	130-113-142	1:100
CD33	FITC	Miltenyi biotech	130-113-348	1:100
CD34	PE-Cy7	BD Biosciences	348811	1:100
CD4	Vioblue	Miltenyi biotech	130-113-258	1:100
CD45	APC	Miltenyi biotech	130-110-633	1:100
CD7	VioBright-FITC	Miltenyi biotech	130-105-844	1:100
CD8	APC-Cy7	BD Biosciences	555367	1:100

Table S4: Antibody used for flow cytometry studies. List of antibodies used to analyze cell subsets from PBMC samples as well as the human HSC reconstitution in PB and BM of mice.

Antigen	Fluorochrome	Supplier	Reference	Dilution
CD11b	APC-Cy7	BD Biosciences	557657	1:100
CD11c	APC	BD Biosciences	550261	1:100
CD223	BUV395	BD Biosciences	745693	1:100
CD25	BV605	BD Biosciences	563061	1:100
CD4	VioBlue	Miltenyi biotech	130-121-132	1:100
CD69	PE-Cy7	BD Biosciences	552879	1:100
CD8	PerCP	BD Biosciences	553036	1:100
F4/80	FITC	Miltenyi biotech	130-117-509	1:100
F4/80	PE	Miltenyi biotech	130-102-422	1:50
FoxP3	PE	BD Biosciences	560408	1:100
Gr1	PerCP-Vio700	Miltenyi biotech	130-102-171	1:50
GzB	FITC	eBioscience	11-8898-82	1:100
IL17	PE-Cy7	Biolegend	506921	1:100
IL2	PE	BD Biosciences	554428	1:100
Ki67	BV605	BD Biosciences	567122	1:100
PD1	VioBlue	Miltenyi biotech	130-121-437	1:100
TNFα	BV510	Biolegend	506339	1:100

Table S5: Antibody used for flow cytometry studies. List of antibodies used to analyze the different immune cells as well as the T8 lymphocytes activation *ex vivo*, on the EL4 lymphoma model.
