# Supplementary figures and images for: Valrubicin-loaded immunoliposomes for specific vesicle-mediated cell death in the treatment of hematological cancers
Source: Cell Death Dis. 2024 May 11;15(5):328. doi: 10.1038/s41419-024-06715-5 (PMC11088660; doi:10.1038/s41419-024-06715-5)

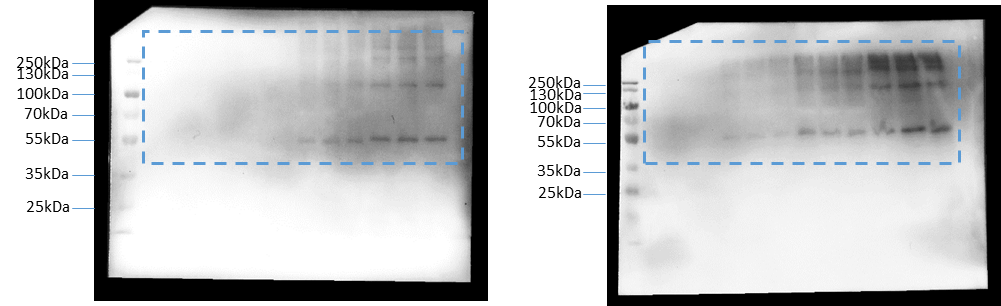


**Full length uncropped western blots corresponding to Figure S1A.**

Supplement: Supplementary file 2 — Original data [file 41419_2024_6715_MOESM2_ESM.docx]
